# Toughening Healable Supramolecular Double Polymer Networks Based on Hydrogen Bonding and Metal Coordination

**DOI:** 10.1002/chem.202402511

**Published:** 2024-11-09

**Authors:** Ilaria Onori, Georges J. M. Formon, Christoph Weder, José Augusto Berrocal

**Affiliations:** ^1^ Adolphe Merkle Institute University of Fribourg Chemin des Verdiers 4 1700 Fribourg Switzerland; ^2^ NCCR Bio-Inspired Materials University of Fribourg 1700 Fribourg Switzerland; ^3^ Institute of Chemical Research of Catalonia (ICIQ) Barcelona Institute of Science and Technology (BIST) Avda. Països Catalans 16 E- 43007 Tarragona Spain

**Keywords:** Double polymer networks, Supramolecular motifs, Healable materials, Improved elasticity, Orthogonal stimuli-responsiveness

## Abstract

Double polymer networks (DNs) consist of two interpenetrating polymer networks and can offer properties that are not merely a sum of the parts. Here, we report an elastic DN made from two supramolecular polymers (SMPs) that consist of the same poly(*n*‐butyl acrylate) (BA) backbone. The two polymers feature different non‐covalent binding motifs, which form dynamic, reversible cross‐links. The polymers were prepared by reversible addition−fragmentation chain‐transfer polymerization of *n*‐butyl acrylate and either the self‐complementary hydrogen‐bonding motif 2‐ureido‐4[1H]pyrimidinone, or the 2,6‐bis(1′‐methylbenzimidazolyl)pyridine ligand, which forms complexes with metal ions. The supramolecular DN made by these components combines features of the single networks, including high thermal stability and resistance to creep. The DN further exhibits excellent healability and displays a higher extensibility and a higher toughness than its constituents. The mechanical characteristics of the DN can be further enhanced by selectively pre‐stretching one of the networks, which is readily possible due to the reversible formation of the supramolecular cross‐links and their orthogonal stimuli‐responsiveness.

## Graphical abstract

We report supramolecular polymer double networks (DNs) based on poly(*n*‐butyl acrylate)s featuring orthogonally responsive 2‐ureido‐4[1H]pyrimidinone (UPy) and 2 : 1 complexes between 2,6‐bis(1′‐methylbenzimidazolyl)pyridine (MeBip) ligand and Zn^2+^ ions as cross‐links. The supramolecular DN exhibits thermal stability, creep resistance, excellent healability, and improved elongation and toughness.

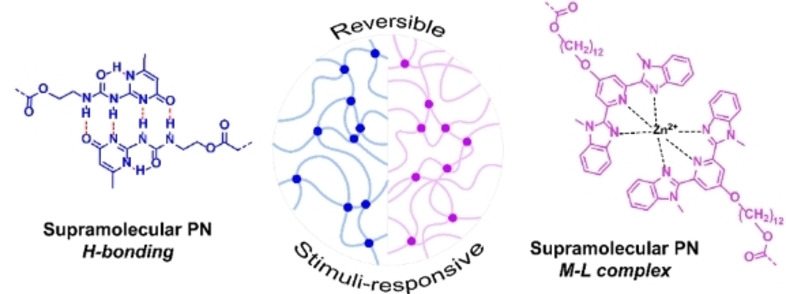

## Introduction

Double polymer networks (DNs) are made by the combination of two polymer networks (PNs) with different properties.^[1]^ These materials are often comprised of a stiffer, more highly cross‐linked network and a softer, more loosely cross‐linked polymer. This combination affords tough materials, whose properties are governed by the fact that one of the networks can act as a sacrificial component, which can dissipate mechanical energy and reinforce the material by fracturing its bonds under mechanical load.^[2]^ This approach was first introduced by Gong *et al*.,^[3]^ who demonstrated that DN hydrogels based on two covalent networks show much higher mechanical strength and toughness than conventional hydrogels.[^2^, ^4^, ^5^] In the meantime, the field has significantly expanded. Relevant to the present work, several examples of DN hydrogels leveraging covalent and supramolecular cross‐links simultaneously, i. e., hybrid DNs, or only supramolecular cross‐links, i. e., supramolecular DNs, have been reported.[^6^, ^7^, ^8^, ^9^, ^10^, ^11^, ^12^, ^13^, ^14^, ^15^, ^16^, ^17^] Moreover, the DN approach has been expanded to solvent‐free elastomers. Such materials were first reported by Creton and co‐workers, who devised highly elastic, poly(acrylate)‐based materials with enhanced stiffness and toughness.^[18]^

While covalent DNs exhibit attractive mechanical properties, these materials cannot be easily repaired or recycled. This limitation is rooted in the *irreversible* nature of covalent bond cleavage, i. e., the sacrificial bonds cannot be reformed at a low energetic cost once broken. One approach to overcome this limitation consists in the preparation of hybrid DNs.[^18^, ^19^, ^20^, ^21^, ^22^, ^23^] Solid hybrid DNs have been shown to possess both remarkable mechanical properties and responsiveness.[^19^, ^24^, ^25^] The former derives from the covalent network, which provides stability and robustness, while the latter is a consequence of the presence of the non‐covalent network (hydrogen bonding, π‐π stacking, or host‐guest interactions), which guarantees tunability and adaptability.^[1]^ For example, some of us reported a DN based on a covalently cross‐liked poly(*n*‐butyl acrylates) and a corresponding network in which 2‐ureido‐4[1H]pyrimidinone (**UPy**) hydrogen dimers serve as reversible cross‐links.^[23]^ The DN exhibited high thermal stability and creep resistance, as well as good extensibility and healing.^[23]^ Lin *et al*.^[26]^ prepared tough nanocomposites consisting of the thermoplastic elastomer poly(styrene‐*block‐*butadiene‐*block*‐styrene) (SBS) and cellulose nanocrystals (CNC). The first network, formed mainly by the CNC nanoparticles, relied on hydrogen bonding interactions between the hydroxyl groups on the filler surface, while the second network was established by cross‐linking the SBS. Additional covalent bonds between the SBS and the chemically modified ends of the CNCs were introduced by thiol‐ene click chemistry. The resulting DN showed good healing and improved mechanical properties compared to the neat SBS.^[26]^ Jiang *et al*. developed a tough, flexible, stretchable, and adhesive DN elastomer based on metal‐ion cross‐linking. The first network was a sulfonated poly(phenoxy) (SPPO) metal‐ion cross‐linked network, while the second was a covalently cross‐linked poly(ethyl acrylate) (PEA) network.^[27]^ The resulting elastomer shows improved energy dissipation, with strain at break of up to 200 %, and was also healable, compared to the covalently cross‐linked network. Finally, Mareliati *et al*. studied a DN made by the combination of a covalently cross‐linked styrene‐butadiene rubber and metal‐ligand interactions between a multidentate ligand and a Zn^2+^ salt.^[22]^ The metallo‐supramolecular interactions conferred improved toughness, strain at break, and tensile strength to the DN.

While numerous solid DNs comprising only covalent or both covalent and supramolecular cross‐links have been investigated, less attention has been dedicated to solid‐state DNs containing only non‐covalent cross‐links.^[28]^ In supramolecular polymer networks, the absence of covalent bonds and the reversible nature of non‐covalent interactions allow for dynamic rearrangements, endowing the material with self‐healing, shape memory, and stimuli‐responsiveness.[^29^, ^30^, ^31^] These networks can reversibly disassemble and reassemble in response to external stimuli, enabling them to adapt to changing environmental conditions.[^32^, ^33^, ^34^, ^35^, ^36^, ^37^, ^38^] Thus, tough supramolecular DNs can overcome the limitations of hybrid DNs, which possess enhanced mechanical strength and toughness, but are not fully recyclable/reprocessable due to the presence of the covalent bonds. Engineering supramolecular interactions at the level of the cross‐links in bulk DNs might also impart several advantages, such as facile processing, tunable properties, full healability, and environmental responsiveness, without sacrificing the strength of traditional DNs.[^39^, ^40^] Finally, supramolecular/dynamic DNs[^37^, ^41^] can show a *continuous* energy dissipation mechanism through the rupture of non‐covalent bonds, with both cross‐links serving as sacrificial bonds, which is fundamentally different and not obtainable with covalent DNs. Weng *et al*.[^42^, ^43^] designed a physically cross‐linked interpenetrating polymer network (IPN) elastomer in which Al‐COO^−^ ion binding was used to construct a first, less dynamic, polymer network with robust cross‐links, and Eu‐iminodiacetate complexes were employed to form a second, more dynamic network structure. This design afforded a strong, self‐healing IPN elastomer. Previous studies from our group reported DNs based on a poly(propylene oxide) (PPO) core featuring two supramolecular PNs. The **UPy** hydrogen‐bonding motif, which dimerizes under formation of quadrupole hydrogen bonds,[^44^, ^45^] and the 2,6‐bis(1′‐methylbenzimidazolyl)pyridine (**MeBip**) ligand,[^46^, ^47^] which forms 2 : 1 or 3 : 1 complexes with metal ions, were used as supramolecular cross‐links. The orthogonality of the two supramolecular interactions and the stimuli‐responsiveness of these non‐covalent cross‐links allowed to address double and triple shape‐memory, along with healability.^[48]^ In a different approach, Konkolewicz and coworkers studied dynamic DNs containing two exchangeable cross‐links. The first network, which served as the sacrificial one, leveraged the hydrogen bonds formed between the self‐complementary **UPy** groups. The second network contained a furan‐maleimide Diels‐Alder adduct as a dynamic covalent cross‐link, instead.[^49^, ^50^, ^51^] These materials were reported to heal under thermal treatment and showed an improvement in fracture energy (from 1.9–2.8 kJm^−2^) compared to the SN elastomers.

Supramolecular DNs have not been extensively studied, but these materials have the potential to combine the structural integrity of traditional polymer networks with the properties derived from the dynamic and reversible nature of supramolecular interactions.[^33^, ^34^, ^52^] Here, we report a supramolecular DN elastomer based on poly(*n*‐butyl acrylate) (**PBA**) and two different non‐covalent cross‐links. We combine the self‐complementary **UPy** hydrogen‐bonding motif and the **MeBip** ligand, which forms complexes with metal ions. The selected motifs are dynamic, reversible, and *orthogonally responsive* to temperature variations and competitive ligands (chemical stimulus), which permits the selective or preferred disassembly of one cross‐link type over the other. The orthogonality of the supramolecular motifs allows to target the selective healing of one network in the presence of the other, which consequently allows to heal first one network, and then the other.

## Results and Discussion

### Design of Materials, Synthesis, Characterization

The two single networks were synthesized using the same polymer core to suppress (micro)phase separation upon DN formation. Poly(*n*‐butyl acrylate) (**PBA**) was chosen as backbone due to its amorphous nature and its low glass‐transition temperature (*T*_g_) of −44 °C, i. e., features that should render the resulting DNs rubbery. The two single supramolecular polymer networks were designed to comprise two distinct, non‐covalent cross‐links. The self‐complementary **UPy** motif[^44^, ^45^, ^53^] was selected to access a polymer network whose cross‐links are based on H bonding (**PBA‐UPy**),[^54^, ^55^, ^56^] while **MeBip**,[^57^, ^58^, ^59^, ^60^, ^61^, ^62^] which forms 2 : 1 complexes with Zn^2+^ ions, was used as the basis for the second supramolecular polymer network (**PBA‐MeBip⋅Zn^2+^**).[^63^, ^64^, ^65^, ^66^]

**Figure 1:**
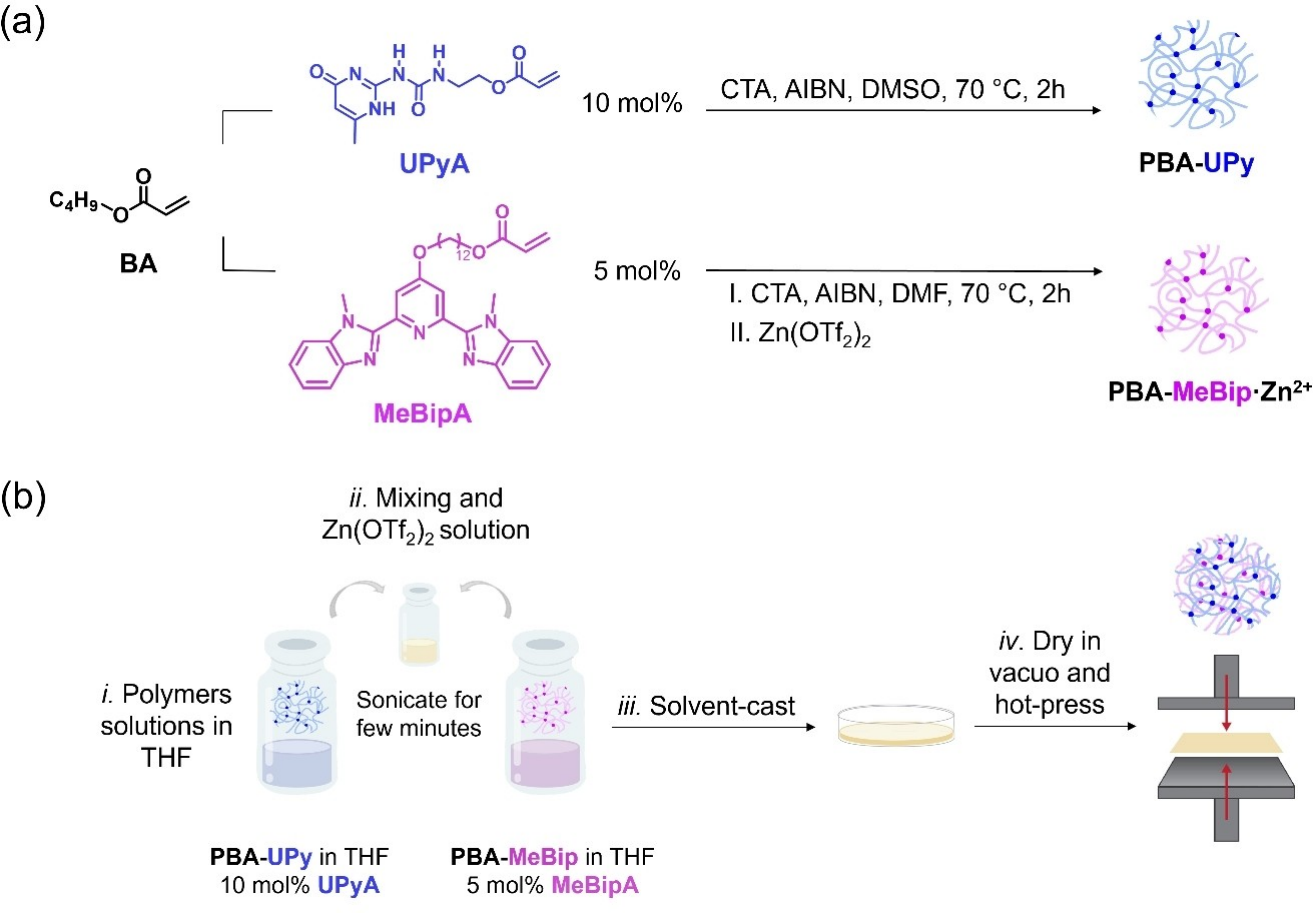
(a) Syntheses of **PBA‐UPy** and **PBA‐MeBip** by RAFT polymerization of **BA** and **UPyA** (10 mol %) or **BA** and **MeBipA** (5 mol %). The **PBA‐MeBip⋅Zn^2+^** network is obtained upon the addition of Zn(OTf_2_)_2_ to a solution of **PBA‐MeBip**. (b) Experimental procedure for the synthesis of **DN**: *i*. **PBA‐UPy** and **PBA‐MeBip** are separately dissolved in THF to produce homogeneous solutions; *ii*. The polymer solutions are combined and a solution of Zn(OTf_2_)_2_ is added; *iii*. The solution is solvent‐cast; *iv*. The resulting material is dried in vacuo and hot‐pressed.

First, we synthesized monomers carrying the supramolecular motifs. **UPyA** was made by reacting 2‐amino‐4‐hydroxy‐6‐methylpyrimidine with 2‐isocyanatoethyl acrylate by adapting an established procedure^[67]^ (for experimental details, see Supplementary Information (SI), page S4). **MeBipA** was synthesized by reacting 12‐((2,6‐bis(1‐methyl‐1H‐benzo[d]imidazol‐2‐yl)pyridin‐4‐yl)oxy)dodecan‐1‐ol (**MeBip‐O‐C_12_‐OH**), with acryloyl chloride, again adapting an established procedure^[63]^ (for experimental details, see page S6). As we did not introduce any modifications to the “classical” designs of the **UPy** and **MeBip** motifs, we speculate that their binding constants in **PBA‐UPy** and **PBA‐MeBip** do not differ significantly from those reported previously.[^45^, ^58^, ^68^, ^69^] Polymers **PBA‐UPy** and **PBA‐MeBip** were both synthesized by reversible addition‐fragmentation chain‐transfer (RAFT) polymerization, using *n*‐butyl acrylate (**BA**) as the main monomer, cyanomethyl dodecyl trithiocarbonate as the chain‐transfer agent (CTA), azobisisobutyronitrile (**AIBN**) as the thermal initiator, and either **UPyA** (10 mol %, corresponding to a nominal cross‐link density of 5 mol %), or **MeBipA** (5 mol %, corresponding to a nominal cross‐link density of 2.5 mol %). We note that due to thermodynamic (binding equilibria) and kinetic (intramolecular binding) effects, the actual cross‐link densities are lower than the nominal values,^[70]^ and to compensate for the lower binding constant of **UPy**[^45^, ^58^, ^68^, ^69^] we used this motif in a higher concentration. The polymerization reactions were carried out at 70 °C (**Figure** 1 **a**), and the polymers were isolated by precipitation after a reaction time of 2 hours. Further purification by repeated precipitation and dialysis afforded **PBA‐UPy** and **PBA‐MeBip**, which were characterized by ^1^H‐ and ^13^C‐NMR spectroscopy (SI, **Figures** **S14–S15** and **S22‐S23**). The integration of pertinent NMR signals shows that the molar fraction of the **UPyA** residues in the polymer is the same as the one in the monomer feed. The number‐average molecular weight (*M_n_*) of 52 kg mol^−1^ and dispersity (*Đ*) of 1.2 of **PBA‐UPy** were determined by size exclusion chromatography (SEC) in THF, while the conversion of 74 % was determined by analysis of ^1^H‐NMR spectra (for details see page S4–5 in the SI). The polymerization of **PBA‐MeBip**, which was characterized by the same methods as **PBA‐UPy**, proceeded in a similar manner. The molar fraction of the **MeBipA** residues in the polymer is again the same as the one in the monomer feed, while *M_n_* (30 kg mol^−1^) and conversion (63 %) were slightly lower than for **PBA‐UPy**. The low dispersity (*Đ*=1.2) reflects that also this polymerization is controlled (for details see page S6–7 in the SI). The supramolecular polymer network **PBA‐MeBip⋅Zn^2+^** was produced by the addition of a Zn(OTf_2_)_2_ stock solution in CH_3_CN/MeOH (9 : 1) to a CHCl_3_ solution of **PBA‐MeBip** and the formation of the 2 : 1 **MeBip:Zn^2+^** complexes was assessed by titration that was monitored by UV‐Vis absorption spectroscopy (**Figure** **S11**).

Films of the individual supramolecular networks **PBA‐UPy** and **PBA‐MeBip⋅Zn^2+^** were then prepared by casting CHCl_3_ solutions of the polymers into Petri dishes (adding a stoichiometric amount of the Zn(OTf_2_)_2_ solution in the case of **PBA‐MeBip**), followed by solvent evaporation and drying in vacuo. Compression‐molding (60 °C or 120 °C, 3 tons, respectively for **PBA‐UPy** and **PBA‐MeBip⋅Zn^2+^**) of the solvent‐cast materials afforded homogeneous films with a thickness of ~250 μm.

The supramolecular DN was prepared in a similar manner by combining solutions of **PBA‐UPy** and **PBA‐MeBip** (**1 : 1 w/w**), followed by the addition of the Zn(OTf_2_)_2_ solution, and drying. The DN was prepared as 1 : 1 w/w ratio of the two supramolecular PNs, as other previously reported systems.[^49^, ^51^, ^71^] Compression‐molding (120 °C, 3 tons) of the solvent‐cast material resulted in homogeneous films with a thickness of ~250 μm (**Figure** 1 **b**). For simplicity, the material thus made, **DN‐UPy‐MeBip⋅Zn^2+^**, is hereafter referred as **DN**. All **DN** films were highly transparent, although with a yellowish hue (**Figures** **S1** and **S2**).

### Thermal and Mechanical Properties

The thermal and thermomechanical properties of **PBA‐UPy**, **PBA‐MeBip⋅Zn^2+^**, and **DN** were characterized by thermogravimetric analysis (TGA), differential scanning calorimetry (DSC), dynamical mechanical analysis (DMA), tensile tests, and creep recovery experiments. The key data extracted from these analyses are compiled in **Table** 1.

**Table 1:** Mechanical properties of the supramolecular polymer networks **PBA‐UPy** and **PBA‐MeBip⋅Zn^2+^**, and the supramolecular double network **DN**.^[a]^

	Storage Modulus *E* ^’^ (MPa)^[b]^ (−70 °C)	Storage Modulus *E* ^’^ (MPa)^[b]^ (25 °C)	Glass Transition Temperature *T*_g_ (°C)^[b]^	Failure Temperature *T*_f_ (°C)^[b]^	Young's Modulus *E*_t_ (MPa)^[c]^	Stress at break *σ*_γ_ (MPa)^[c]^	Elongation at break *ϵ*_β_ (%)^[c]^	Glass Transition Temperature *T*_g_ (°C)^[d]^
**PBA‐UPy**	2192±274	4.6±0.6	0.4	109	2.9±0.2	2.3±0.2	109±11	−33
**PBA‐MeBip⋅Zn^2+^**	1940±118	11±1.0	−8	180	9.0±0.3	2.9±0.3	91±12	−38
**DN**	1973±21	5.5±0.3	−3	152	4.9±0.2	2.3±0.2	180±20	−36

[a] Data represent averages of n=3–8 individual measurements±standard deviation. [b] Measured by DMA with a heating rate of 3° min^−1^. [c] Measured by tensile tests at 25 °C with a strain rate of 150 % min^−1^. [d] Measured by DSC with a heating rate of 10° min^−1^.

**Figure 2:**
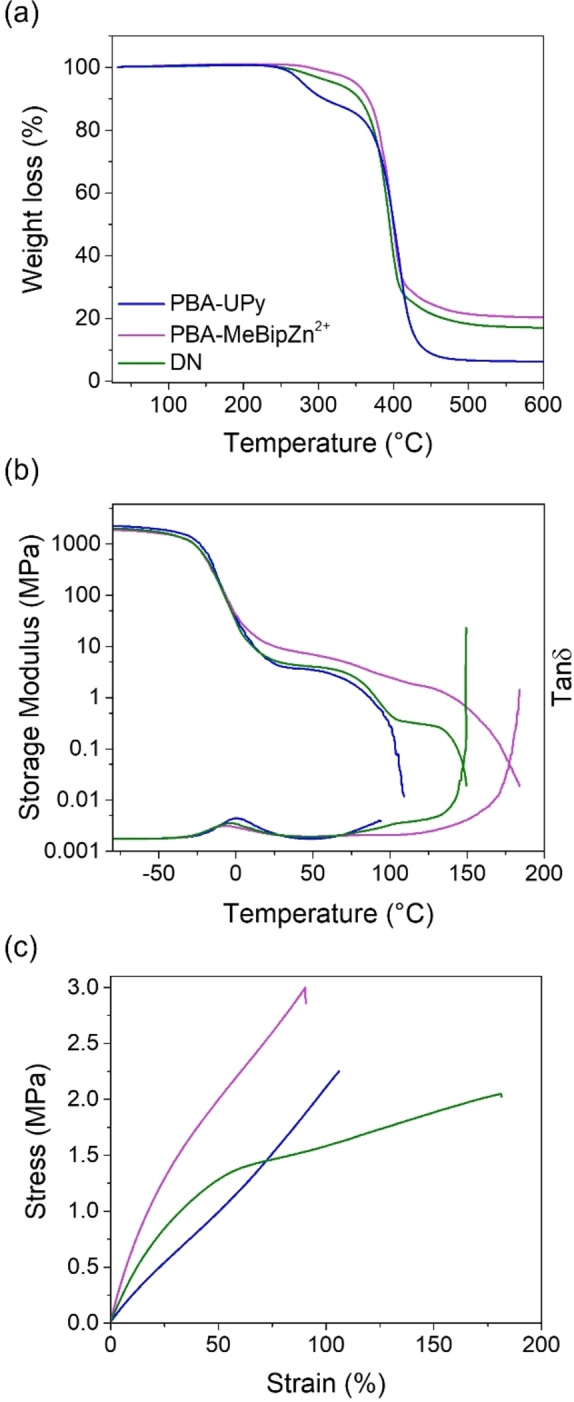
Thermal and mechanical characterization of **PBA‐UPy**, **PBA‐MeBip⋅Zn^2+^**, and **DN**. The panels show (a) thermogravimetric analysis (TGA) traces, (b) dynamic mechanical analysis (DMA) traces, and (c) stress−strain curves. The heating rates for the TGA and DMA experiments were 10 and 3 °C min^−1^, respectively, and tensile tests were carried out at ambient temperature and a strain rate of 150 % min^−1^.

TGA was performed to probe the thermal stability of the three networks (**Figure** 2 **a**, SI **Figure** **S2**). The TGA trace of **PBA‐UPy** shows two consecutive steps, a smaller one (10 % of weight loss), with onset at ~200 °C and a second, more pronounced one, above ~300 °C. This behavior has been observed previously for other, similarly structured **UPy**‐containing polymer systems; the first weight‐loss step is associated with the elimination of the pendant **UPy** groups (10 wt % loading in our system), and the second to the degradation of the polymer backbone (which is system‐specific).[^72^, ^73^] The TGA trace of **PBA‐MeBip⋅Zn^2+^** reveals an onset of weight loss at 250 °C, due to the higher thermal stability imparted by the metal‐ligand coordination, as previously observed by our group.^[48]^ The TGA trace of **DN** shows features of the two constituent single networks.

The glass transition temperature (*T_g_*) of all **PBA**‐based materials was determined by DSC (**Figure** **S3**). The second heating trace of **PBA‐UPy** shows a glass transition at −33 °C, whereas the *T_g_* of **PBA‐MeBip⋅Zn^2+^** is observed at a slightly lower temperature (−38 °C), consistent with the lower density of cross‐links. **DN** exhibits a *T_g_* of −36 °C, in between those of the individual components. Importantly, this provides a first indication that the two individual networks are not phase‐separated. None of the DSC traces shows thermal transition related to the melting of either a **UPy** or a **MeBip** hard phase, suggesting the absence of microphase‐separated, crystalline domains of these aromatic moieties in both single and double networks.^[48]^ This conclusion is supported by small‐ and wide‐angle X‐ray scattering (SAXS‐WAXS) measurements (**Figure** **S5**). The SAXS region displays two broad halos at q=0.9 nm^−1^ and q=5.0 nm^−1^, which are not associated with periodic morphologies present in the samples (**Figure** **S5**). The WAXS region features a broad, structureless halo at q=14.0 nm^−1^, instead, which is further indicative of a fully amorphous material (**Figure** **S5**).

**Figure** 2 **b** shows the DMA traces of **PBA‐UPy**, **PBA‐MeBip⋅Zn^2+^**, and **DN**. At −70 °C, i. e., below *T*_g_, all materials display a storage modulus (*E*′) around 2 GPa. The DMA trace of the **PBA‐UPy** network displays the transition from a glassy to a rubbery polymer around a *T_g_* of 0 °C, which is indicated by a local maximum of the tanδ trace.^[23]^ The difference in the *T*_g_ values established by DSC and TGA is typical and related to the difference in techniques (**Table** 1). At 25 °C, in the rubbery regime, **PBA‐UPy** shows a storage modulus of 4.6 MPa, the lowest of the three materials, in accordance with the lower association constant of the **UPy** groups compared to the M−L interactions.^[69]^ Above ca. 50 °C, the tanδ trace of **PBA‐UPy** starts to increase, while *E’* drops, due to the gradual dissociation of the **UPy** dimers. The sample eventually fails at ca. 109 °C.

The DMA trace of **PBA‐MeBip⋅Zn^2+^** shows this polymer transitions from the glassy to the rubbery state at a *T_g_* of −8 °C (tanδ peak), i. e., at a slightly lower temperature than **PBA‐UPy**, as also observed by DSC. Above the glass transition, the material shows a broad rubbery plateau. At 25 °C, *E*′ assumes a value of 11 MPa, which is the highest of the reported materials and reflects that the M−L complexes form robust cross‐links. The material also shows the highest failure temperature (180 °C), although the DMA trace shows that *E*’ drops across the entire rubbery plateau, indicative of gradual dissociation of the dynamic M−L complexes.

The DMA trace of **DN** shows a *T_g_* of −3 °C, between the values of the two single networks. The tanδ peak does not appear to be significantly broadened, which is again indicative of the absence of phase separation. At 25 °C the DMA shows an *E*’ value of 5.5 MPa, between the values of the two single networks, although it is noted that the value is closer to the one of **PBA‐UPy**. This is consistent with the fact that the fraction of supramolecular motifs that form cross‐links decreases with their concentration.^[70]^ The DMA trace of **DN** shows two distinct drops in *E*’ that are related to the dissociation of the different cross‐links. The first transition, below 100 °C, is associated with the dissociation of the **UPy** dimers, while the second step sets in just below 150 °C and is associated with the M−L dissociation. **DN** fails at 152 °C, i. e., at a lower temperature than the neat metallosupramolecular network, which is consistent with the lower density of M−L complexes. Overall, the thermomechanical properties of **DN** appear to follow a rule of mixture and reflect the expected contributions of the of two single networks.

The mechanical properties of the three materials were further characterized by uniaxial tensile tests that were carried out at ambient temperature (25 °C) with a strain rate of 150 % min^−1^ (**Figure** 2 **c**, **Table** 1). All materials show largely linear stress‐strain curves. In accordance with the *E*’ values determined by DMA, **PBA‐UPy** displays the lowest Young's modulus (*E*_t_=2.9 MPa). The polymer also features the lowest stress at break (*σ*_γ_=2.3 MPa), while its strain at break *ϵ*_β_ of 109 % is appreciable. The Young's modulus (*E*_t_=9.0 MPa) and stress at break (*σ*_γ_=2.9 MPa) of **PBA‐MeBip⋅Zn^2+^** are the highest of the materials reported, which is consistent with the DMA data and the fact that metal‐ligand interactions are stronger than hydrogen bonding interactions. The strain at break (*ϵ*_β_=91 %) of **PBA‐MeBip⋅Zn^2+^** is slightly lower than that of **PBA‐UPy**, although the difference is not statistically significant (**Table** 1). While **DN** shows *E*_t_ (4.9 MPa) and *σ*_γ_ (2.3 MPa) values that fall again between those of the two single networks, this material exhibits significantly higher extensibility (*ϵ*_β_=180 %) and also higher toughness (2814 kJm^−3^) than the two constituting materials (**Table** **S1**). The enhanced toughness is consistent with the disassembly/re‐assembly of the **UPys** bonds, which act as sacrificial bonds and rupture under load, dissipating energy. The stress‐strain curve of **DN** shows a change in slope at ca. 50 % strain, which is consistent with the disassembly of the **UPy** dimers and a reduction in the density of cross‐links in the material.

The materials were further characterized by creep recovery experiments. The samples were kept under a constant load (0.01 MPa) for 15 minutes and then allowed to relax for another 15 minutes (**Figure** **S6**). The creep recovery was investigated at 25 °C and 60 °C. At room temperature, **PBA‐UPy** shows a small extent of deformation, with a strain that remains almost constant at 1.2 %. This material fully recovers and relaxes back to the initial strain once the stress is released (the load was removed instantly), reflecting that at 25 °C the **UPy**‐based cross‐links are not very dynamic. At 60 °C, the material creeps considerably and deforms to a strain of 22 % after 15 min in a manner that depends linearly on time. When the stress is removed, hardly any relaxation (final strain=18 %) is observed. This behavior is consistent with the highly dynamic binding between the **UPy** motifs at this temperature, which leads to irreversible deformation.^[23]^ The extent of creep observed for **PBA‐MeBip⋅Zn^2+^** is much smaller, with strains of 0.3 % and 0.9 % observed at 25 °C and 60 °C, respectively. At both temperatures, the original strain is fully recovered, which is indicative of the highly robust nature of the M−L cross‐links in this temperature range. Intriguingly, the creep characteristics of **DN** are dominated by the more robust metallosupramolecular network. At 25 °C, the creep profile mirrors the ones of the two single networks, with strain values that remained quite constant during both load (~0.5 %) and recovery (~0.3 %). At 60 °C, the material shows a somewhat higher deformation than **PBA‐MeBip⋅Zn^2+^** (5.0 % compared to 1.0 %), albeit it was still far from the deformation observed for **PBA‐UPy** (22 %). The relative extent of recovery upon stress removal is also much better than that of **PBA‐UPy**. Thus, **DN** is much less prone to creep than the neat hydrogen‐bonded network, especially at 60 °C, and the properties are much closer to the ones of the metallosupramolecular polymer, thanks to the reinforcement and the stability imparted by the M−L cross‐links.

### Pre‐Stretching One Network

Based on previous works, we expected that pre‐stretching one of the networks would increase the toughness of **DN**, as the propensity of the cross‐links to dissociate and serve as sacrificial bonds that dissipate mechanical energy is increased.[^2^, ^18^] In the case of the present **DN** the preferential, if not exclusive, pre‐stretching of one network type can be achieved by temporarily disassembling the cross‐links of the other network by deforming the material to a given strain, applying a selective external stimulus, and re‐establishing the second network while the strain is still applied (see SI, page **S11**, for experimental procedures, **Figures** **S8‐S9**). To achieve this, we either heated **DN** films to preferentially dissociate the **UPy** cross‐links and pre‐stretch **PBA‐MeBip⋅Zn^2+^** or exposed the samples to tetramethylethylenediamine (TMEDA) vapors to selectively dissociate the metallosupramolecular network and pre‐stretch **PBA‐UPy**. TMEDA serves as a competing ligand that can sequester the Zn^2+^ ions from the **MeBip⋅Zn^2+^** complexes. The application of vacuum allows the removal of TMEDA (boiling point=121 °C at 1 atm) and restores the original **MeBip⋅Zn^2+^** complexes.

In order to select the best experimental conditions for the pre‐stretching of the **PBA‐MeBip⋅Zn^2+^** network at elevated temperature, stress‐relaxation experiments were conducted on the two single networks, **PBA‐UPy** and **PBA‐MeBip⋅Zn^2+^**, and on **DN**. We investigated the reduction of the stress over time (20 minutes) while maintaining a constant strain of 1 % or 60 % at different temperatures (**Figure** **S7**). Based on the DMA data (**Figure** 2 **b**), which reflects that the cross‐links in the **PBA‐UPy** network should be almost fully dissociated at 100 °C, we carried out initial experiments at this temperature. At this temperature, **PBA‐UPy** starts to flow, and therefore, the extensional stress relaxation cannot be measured. The stress of **PBA‐MeBip⋅Zn^2+^** fully relaxes in less than 6 minutes, even under low‐strain conditions (1 %), while **DN** relaxes even faster (**Figure S7a**). Thus, the data clearly show that a temperature of 100 °C is too high for the pre‐stretching experiments, as the M−L network is also rather dynamic under these conditions. We thus lowered the temperature to 60 °C, which marks the onset of the tanδ trace of **PBA‐UPy** that is associated with the dissociation of the supramolecular cross‐links. At a strain of 1 %, the stress relaxation of **PBA‐UPy** is complete in ca. 5 minutes, while **PBA‐MeBip⋅Zn^2+^** is quite stable, showing only about 50 % relaxation in 20 minutes. **DN** relaxes in ca. 15 minutes (**Figure S7b**). When the strain is increased to 60 %, both **PBA‐UPy** and **DN** fully relax in less than 5 minutes. Unfortunately, **PBA‐MeBip⋅Zn^2+^** failed under the same experimental conditions, perhaps because the strain rate was too high (**Figure S7d**). Nevertheless, the data suggest that a temperature of 60 °C is sufficient to dissociate the cross‐links of the **PBA‐UPy** network, while the M−L cross‐links in **PBA‐MeBip⋅Zn^2+^** remain at least partially intact. Considering that **PBA‐MeBip⋅Zn^2+^** films fail at 91 % strain during the tensile experiments at room temperature (25 °C), we performed the same experiments at 60 °C with a strain of 40 % (**Figure S7c**). Gratifyingly, the profile obtained was in line with the data obtained when a strain of 60 % was reached, i. e., **PBA‐MeBip⋅Zn^2+^** does not relax completely.

**Figure 3:**
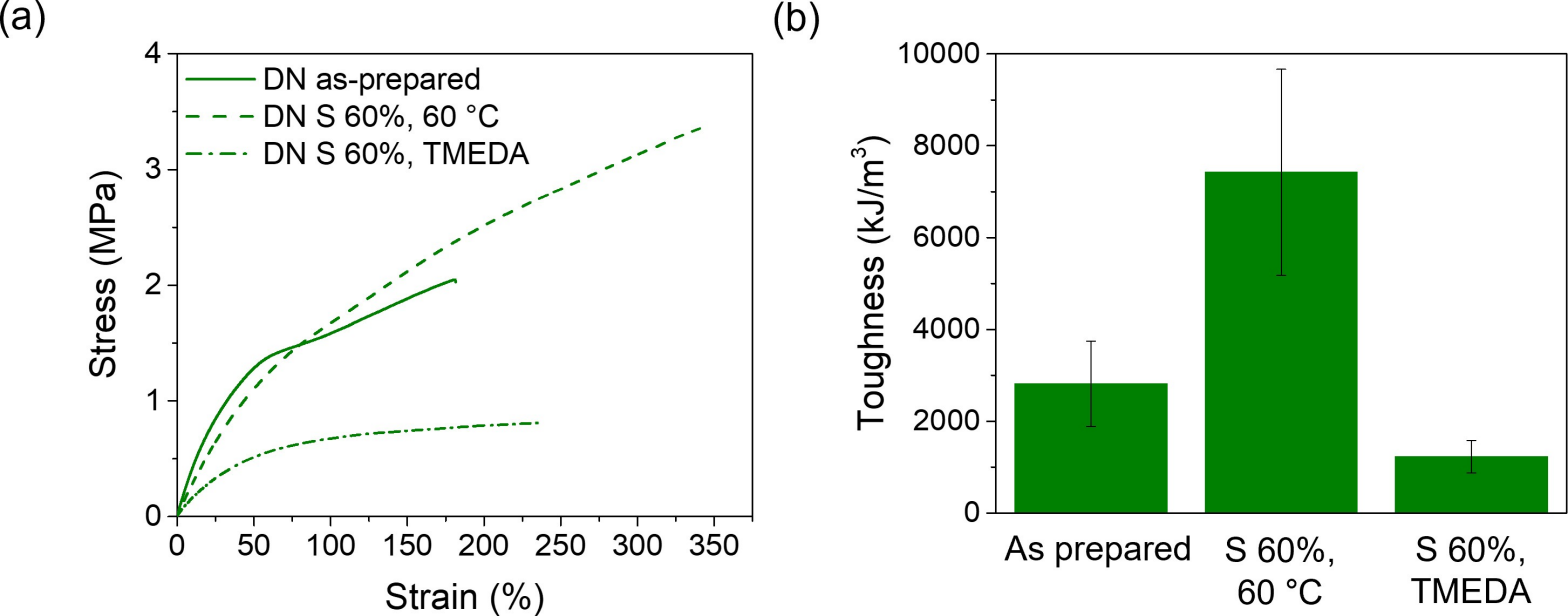
(a) Stress‐strain curves of an as‐prepared film of **DN** (solid line), as well as samples that were pre‐stretched (60 % strain) while heated to 60 °C (dashed line) or exposed to TMEDA vapors (dash‐dotted line). (b) Toughness of the samples reported in (a) (error bars represent the standard deviation).

The thermally assisted pre‐stretching of the metallosupramolecular network within **DN** (see SI, page **S11**, for experimental procedures, **Figure** **S8**) was thus realized by submitting a strip of **DN** to a strain of 60 %, immersing the sample in a water bath kept at 60 °C for 10 minutes, cooling the sample to room temperature, and finally releasing the stress. In addition, we measured the extent of irreversible deformation (see SI, **Figure** **S8**) and observed a residual strain of 56 %±15. We then performed uniaxial tensile tests (25 °C, strain rate=150 % min^−1^) on the pre‐stretched material (**Figure** 3 **a**). After pre‐stretching, *E*_t_ is slightly lower than in the as‐prepared **DN** (3.4 MPa *vs*. 4.9 MPa), but the data show a significant increase in the stress (3.3 MPa vs. 2.3 MPa) and strain at break (350 % vs. 180 %), while the toughness is almost tripled (**Table** 2). We also explored the pre‐stretching of the hydrogen‐bonded **PBA‐UPy** network within **DN** by exposing stretched **DN** films to TMEDA vapors. In this case, the chemically assisted pre‐stretching was realized by submitting a strip of **DN** to a strain of 60 %, exposing the sample to TMEDA vapors for 3 minutes, applying vacuum to remove TMEDA traces, and finally releasing the stress (see page **S11** for experimental procedures, **Figure** **S9**). We performed uniaxial tensile tests (room temperature, strain rate=150 % min^−1^) on the pre‐stretched material (**Figure** 3 **a**). In this case, a residual strain of 33 %±12 was observed (see SI, **Figure** **S9**). However, the tensile tests of the thus‐treated material (**Figure** 3, **Table** 2) reveal a decrease of *E*_t_ and *σ*_γ_ to 2.2 MPa and 0.8 MPa, respectively, compared to 4.9 MPa and 2.3 MPa for the as‐prepared **DN**. While *ϵ*_β_ (240 %) increases slightly vis‐à‐vis the as‐prepared **DN** (180 %), the toughness is reduced to half of its original value (1484 kJm^−3^). Thus, our data reflect that the pre‐stretching of the M−L network at elevated temperature, while the **PBA‐UPy** network is disassembled, is clearly more effective than the orthogonal approach, as this process significantly toughens **DN**. A plausible explanation may lie in the fact that the **UPy** groups are likely more dynamic than the metal‐ligand interactions at room temperature, which allows to re‐establish part of the cross‐links more efficiently. In contrast, the metal‐ligand complexes only reform to a smaller extent (or do not reform at all). The difference between the dynamic character of hydrogen bonding motifs and metal‐ligand complexes is well‐established and has been widely reported in the literature.[^74^, ^75^, ^76^, ^77^]

**Table 2:** Mechanical properties of the as‐prepared **DN**, as pre‐stretched (60 % strain) while heated to 60 °C, and as pre‐stretched (60 % strain) while exposed to TMEDA vapors.^[a]^

	Young's Modulus *E_t_* (MPa)^[b]^	Stress at break *σ*_γ_ (MPa)^[b]^	Elongation at break *ϵ*_β_ (%)^[b]^	Toughness (kJm^−3^)^[c]^
**DN** as prepared	4.9±0.2	2.3±0.2	180±20	2814±930
**DN** pre‐stretched at 60 °C	3.4±0.5	3.3±0.6	350±56	7420±2247
**DN** pre‐stretched in presence of TMEDA	2.2±0.1	0.8±0.1	240±57	1484±360

[a] Data represent averages of n=3–8 individual measurements±standard deviation. [b] Measured by tensile tests at 25 °C with a strain of 150 % min^−1^. [c] Determined by the area under the stress‐strain curves.

### Healing Behavior

Finally, we investigated the possibility of healing defects in **PBA‐UPy**, **PBA‐MeBip⋅Zn^2+^**, and **DN**. Healability is imparted by the dynamicity of the supramolecular interactions. The orthogonal responsiveness of the two supramolecular networks allows addressing the two components separately, which makes it possible to repair the two networks sequentially, and thus heal **DN** while a mechanical load is applied.

**Figure 4:**
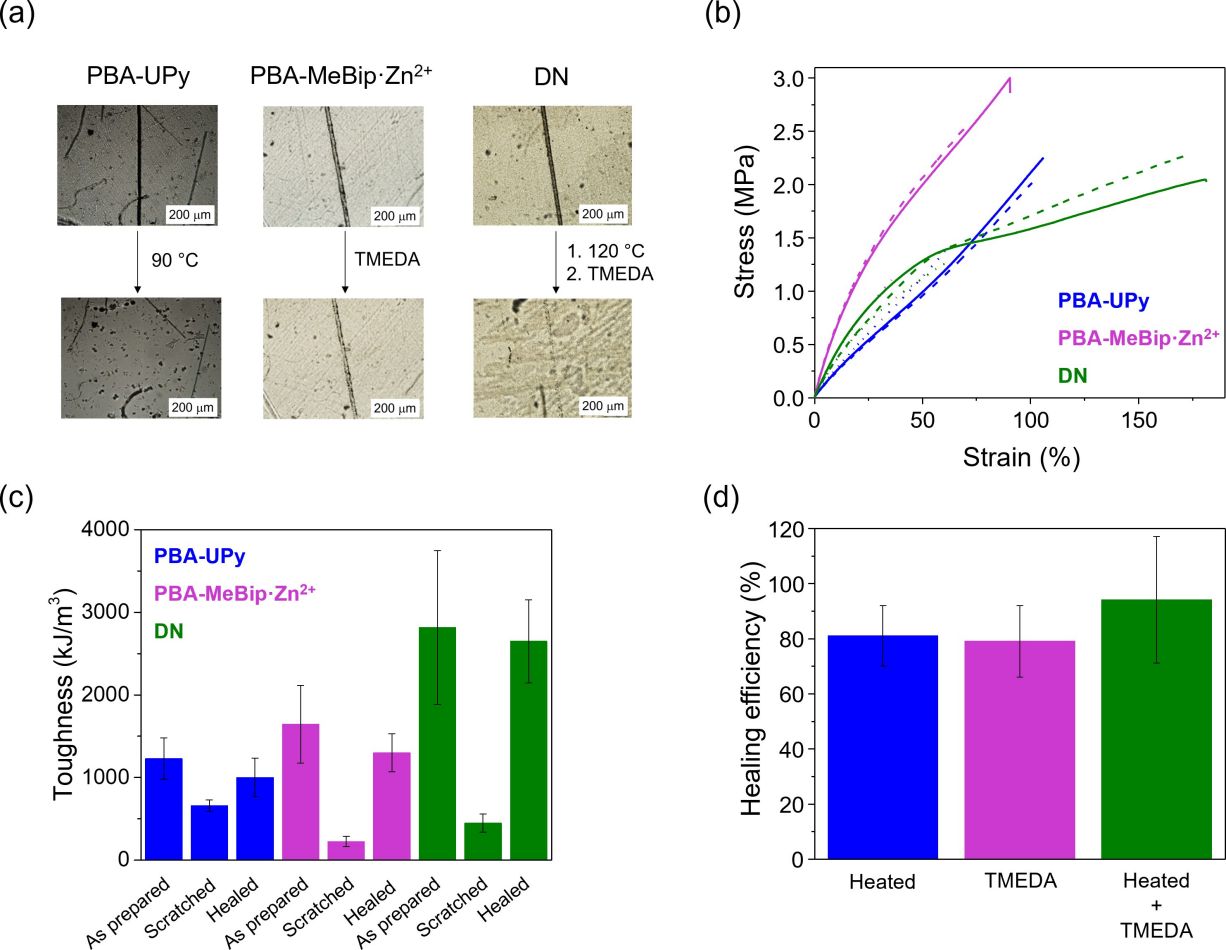
(a) Optical micrographs illustrating the healing of **PBA‐UPy**, **PBA‐MeBip⋅Zn^2+^**, and **DN**. Samples were scratched to a depth of ~50 % and then healed by (i) heating to 90 °C for 40 min, (ii) exposure to TMEDA vapors for 5 min, and (iii) heating to 120 °C for 90 min and subsequent exposure to TMEDA vapors for 5 min (scale bar=200 μm). (b) Stress‐strain curves of the samples shown in (a) in the as‐prepared (solid lines), scratched (dotted lines), and healed (dashed lines) state. (c) Toughness of the samples shown in (a,b), expressed by the area under the stress‐strain curves shown in (b). (d) Healing efficiency achieved for the different materials and protocols. The healing efficiency is expressed as the ratio between the toughness of healed and as‐prepared material. All error bars represent the standard deviation.

Films of each material were intentionally scratched with a razor blade to a depth corresponding to ca. 50 % of the film thickness. The healing process was then qualitatively assessed, by taking optical microscopy (OM) images before and after the healing (**Figure** 4 **a**). **PBA‐UPy** was healed by heating the scratched material at 90 °C for 40 minutes. We found that these conditions, which were optimized for **PBA‐UPy** in a previous study from our group,^[23]^ afforded excellent results also in this case. The sample was then cooled back to room temperature and the healing efficiency was quantitatively assessed by tensile tests. **PBA‐UPy** is able to efficiently heal at that temperature, as already previously reported.^[23]^ Indeed, the scratch fully disappears (**Figure** 4 **a**) due to the dynamic and reversible nature of the supramolecular cross‐links. **PBA‐MeBip⋅Zn^2+^** was healed by exposing a film of the scratched material to TMEDA vapors for 5 minutes, and then vacuum was applied to remove TMEDA traces. This treatment allowed the cut to disappear almost completely (**Figure** 4 **a**). Finally, we investigated the healability of **DN**, by applying the two stimuli sequentially. **DN** was first heated to 120 °C for 90 min to selectively heal the **PBA‐UPy** network, and then treated with TMEDA vapors for 5 minutes to heal **PBA‐MeBip⋅Zn^2+^**. As it is evident from the picture shown in **Figure** 4 **a**, the cut almost disappeared after this treatment. It should be stressed that both the supramolecular motifs used as cross‐links need high temperatures to induce healing, as they are not sufficiently dynamic at room temperature. This characteristic hinders self‐healing or healable behavior at room/low temperature for **PBA‐UPy** and **PBA‐MeBip⋅Zn^2+^**, but it also makes these materials (and the resulting **DN**) resistant to creep.

The healing of the three materials was further quantified by tensile tests of the as‐prepared, scratched, and healed samples, and the healing efficiency was evaluated by comparing the toughness of the healed and the as‐prepared samples (**Figures** 4 **b–d**, **Figure S10a–c** and **Table** **S1**). The scratched samples exhibit a substantially reduced elongation and stress at break and, consequently, a much lower toughness than the as‐prepared materials. Gratifyingly, these characteristics can be fully, or at least largely, restored. When **PBA‐UPy** was healed by thermal treatment in optimized conditions, a healing efficiency (ratio between the toughness of the healed and the as‐prepared material) of 81 % was observed. Treatment of scratched **PBA‐MeBip⋅Zn^2+^** with TMEDA vapors resulted in a healing efficiency of 79 %. In the case of **DN**, the healing efficiency was evaluated for different conditions, namely after only heating (120 °C, 90 min, 67 %), after exposing the samples only to TMEDA vapors (57 %), and, finally, after the successive application of both these stimuli, which resulted in a healing efficiency of 94 % (**Figure** 4 **d**, **Table** **S1**).

These data demonstrate that all the investigated materials show excellent healing efficiency, and the sequential application of thermal and chemical stimuli allows **DN** to almost completely regain its mechanical properties. The data are in line with previous studies conducted on materials containing the same supramolecular cross‐links.^[48]^

## Conclusions

In summary, we have prepared elastomeric supramolecular polymer networks based on an *n*‐butyl acrylate backbone, featuring either hydrogen bonding groups or metal‐ligand complexes as supramolecular cross‐links. The supramolecular polymer networks were combined to generate the corresponding DN. The DN owes its high thermal stability and creep resistance to the presence of the more robust metal‐ligand interactions but, at the same time, shows enhanced extensibility and toughness, which appear to be imparted by the more dynamic UPy groups. By selectively pre‐stretching one of the networks, it is possible to enhance the extensibility and the toughness of the DN considerably. The dynamic and highly responsive supramolecular interactions allow for excellent healing of all the materials, with a measured healing efficiency of 94 % for the DN due to the synergetic action of thermal and chemical stimuli. Future investigations will focus on understanding the influence of the DN composition, here only investigated in a 1 : 1 weight ratio, (also) on the healing efficiency.

## Conflict of Interests

The authors declare no conflict of interest.

1

## Supporting information

As a service to our authors and readers, this journal provides supporting information supplied by the authors. Such materials are peer reviewed and may be re‐organized for online delivery, but are not copy‐edited or typeset. Technical support issues arising from supporting information (other than missing files) should be addressed to the authors.

Supporting Information

## Data Availability

The data that support the findings of this study are available from the corresponding author upon reasonable request.
