# Supplementary material for: Toughening Healable Supramolecular Double Polymer Networks Based on Hydrogen Bonding and Metal Coordination
Source: Chemistry. 2024 Nov 9;30(72):e202402511. doi: 10.1002/chem.202402511 (PMC11665491; doi:10.1002/chem.202402511)
Supplement: Supplementary file 1 — Supporting Information [file CHEM-30-e202402511-s001.pdf]

Chemistry–A European Journal

Supporting Information

Toughening Healable Supramolecular Double Polymer Networks Based on Hydrogen Bonding and Metal Coordination

Ilaria Onori, Georges J. M. Formon, Christoph Weder,* and José Augusto Berrocal*

SUPPORTING INFORMATION

Toughening Healable Supramolecular Polymer Double Networks Based on Hydrogen Bonding and Metal Coordination

Ilaria Onori[1], Georges J. M. Formon[1,2], Christoph Weder[1]*, José Augusto Berrocal[1,3]*,

[1]Adolphe Merkle Institute, University of Fribourg, Chemin des Verdiers 4, CH-1700 Fribourg, Switzerland. [2] NCCR Bio-Inspired Materials, University of Fribourg, 1700 Fribourg, Switzerland. [3]Institute of Chemical Research of Catalonia, Barcelona Institute of Technology (BIST), Avinguda dels Països Catalans, 16, 43007 Tarragona, Spain.

Email : christoph.weder@unifr.ch, jberrocal@iciq.es,

Table of Contents

General materials and methods	S3-4
Materials	S3
Instrumentations and methods	S3-S4
Synthetic Procedures and Analytical Data	S4
Synthesis of **UPyA**	S4
Synthesis of **PBA-UPy**	S4-S5
Preparation of **PBA-UPy** films	S5
Synthesis of **MeBip-OH**	S5
Synthesis of **MeBip-C_{12}-OH**	S5-S6
Synthesis of **MeBip-A**	S6
Synthesis of **PBA-MeBip**	S6
Preparation of **PBA-MeBip·Zn^{2+}** films	S7
Synthesis of **DN**	S7
Pictures of **DN** films	S8
Transmission spectrum of **DN**	S8
TGA data of **PBA-UPy**, **PBA-MeBip·Zn^{2+}** and **DN** films	S8
DSC data of **PBA-UPy**, **PBA-MeBip·Zn^{2+}** and **DN** films	S9
X-Ray Scattering of **DN**	S9
Creep of **PBA-UPy**, **PBA-MeBip·Zn^{2+}** and **DN** films	S9
Stress-relaxation of **PBA-UPy**, **PBA-MeBip·Zn^{2+}** and **DN** films	S10
Thermo-chemical healing experiments and tensile data	S11-S12

Tensile data, toughness and healing efficiency S13

UV-Vis spectra S14

NMR spectra S15

1H-NMR spectrum of **UPyA** S15

13C-NMR spectrum of **UPyA** S15

1H-NMR spectrum of **PBA-UPy** S16

13C-NMR spectrum of **PBA-UPy** S16

1H-NMR spectrum of **MeBip-OH** S17

13C-NMR spectrum of **MeBip-OH** S17

1H-NMR spectrum of **MeBip-C_{12}-OH** S18

13C-NMR spectrum of **MeBip-C_{12}-OH** S18

1H-NMR spectrum of **MeBip-A** S19

13C-NMR spectrum of **MeBip-A** S19

1H-NMR spectrum of **PBA-MeBip** S20

13C-NMR spectrum of **PBA-MeBip** S20

General materials and methods

Materials

Spectroscopic grade $CHCl_3$ (Acros) and $CDCl_3$ (Sigma Aldrich) were filtered through a plug of dry, activated (Brockman I) basic alumina prior to use to remove acidic impurities. *n*-Butyl acrylate (Sigma Aldrich) was also filtered through a plug of dry, activated (Brockman I) basic alumina to remove the inhibitors prior to polymerization. All other solvents and reagents (Sigma Aldrich) were used as received.

Instrumentations and Methods

NMR spectroscopy. ^{1}H (400.19 MHz) and ^{13}C (100.63 MHz) NMR spectra were recorded at 297.2 K on a Bruker Avance DPX 400 spectrometer in $CDCl_3$ or DMSO-d_6.^{1}H-NMR spectra were calibrated to the residual solvent peak of $CHCl_3$ or DMSO at 7.26 or 2.50 ppm and ^{13}C NMR spectra were referenced against the signal of $CHCl_3$ or DMSO at 77.36 or 39.52 ppm. Data were treated with the MestReNova (12.0.2) software suite and all chemical shifts δ are reported in parts per million (ppm) with coupling constant (*J*) in Hz (multiplicity: s = singlet, bs= broad singlet, d= doublet, dd= doublet of doublets, t = triplet, q= quartet, m = multiplet, br = broad signal).

UV-vis absorption spectroscopy. Solution phase UV-Vis absorption spectra were recorded on a Shimadzu UV-2401 PC spectrophotometer using quartz cuvettes of 1 cm path length.

Size exclusion chromatography (SEC). SEC analyses were performed on an Agilent Technologies 1200 series HPLC system equipped with an Agilent PLgel mixed guard column (particle size = 5 μm) and two Agilent PLgel mixed-D columns (ID = 7.5 mm, L = 300 mm, particle size = 5 μm). THF was used as eluent at a flow rate of 1.0 mL min–1 and a UV detector (Agilent 1200 series, λ = 346 nm), along with Wyatt Technology Corp. (Optilab REX interferometric refractometer and miniDawn TREOS light scattering detector) were used to monitor the signal. Data were processed with Astra software (Wyatt Technology Corp.) and molecular weights (M_n) and dispersity (Đ) were determined based on poly(methyl methacrylate) PMMA standards.

Transmission spectroscopy. Optical microscopy was performed with an Axio Scope.A1 (Zeiss, Germany) light microscope connected to a VIS-LED light source. Images were taken with a 20× objective (Zeiss, EC Epiplan-Apochromat, NA = 0.6, DH DIC) calibrated with a white diffuser. Transmittance spectral data were obtained by placing an optical fiber in a plane confocal to the image plane connected to a spectrometer (OceanOptics, QEPRO). Measurements were calibrated using a glass slide as the light reference, while the dark reference was recorded with the light source covered with a black sheet.

Thermogravimetric Analysis (TGA). Thermogravimetric analyses (TGA) were conducted under N_2 with a Mettler-Toledo STAR system from 25 to 600 °C at a rate of 10 °C min^{-1}.

Differential Scanning Calorimetry (DSC). Differential scanning calorimetry (DSC) measurements were performed under N_2 using a Mettler-Toledo STAR system operating at a heating/cooling rate of 10 °C·min^{-1} from -80 to 200 °C.

X-Ray Scattering. Small-angle X-ray scattering (SAXS) and wide-angle X-ray scattering (WAXS) experiments were performed with a NanoMax-IQ camera (Rigaku Innovative Technologies, Auburn Hills, MI, USA). The camera was equipped with a Cu target sealed tube source (MicroMax 003 microfocus, Rigaku), and the scattering data were recorded by a Pilatus 100 K detector (Dectris). Scattering spectra are presented as a function of the momentum transfer $q = 4\pi\lambda^{-1} \sin(\theta/2)$, where θ is the scattering angle and λ = 0.1524 nm is the photon wavelength.

Compression molding. Polymer films were prepared by compression molding in a Carver CE Press at a temperature of 60 °C or 120 °C and pressure of 3 tons for 20 min (plates size 14.5 cm x 14.5 cm).

Dynamic Mechanical Analysis (DMA). Dynamic mechanical analyses (DMA) were conducted under N_2 on a TA Instruments DMA Q800 with a heating rate of 3 °C·min^{-1}, a frequency of 1 Hz, and an amplitude of 15 µm in the range of −80 to 200 °C, using tensile clamps and rectangular-shaped samples (width: 5.35 mm, thickness: 0.25-0.3 mm), unless indicated otherwise. Reported mechanical data are averages of 3−5 independent experiments, and all errors are standard deviations.

Tensile tests. Tensile measurements were carried out according to ASTM D882, at room temperature, with a static material testing machine from Zwick/Roell equipped with a 200 N Xforce HP load cell. Data are reported as averages of 5-8 independent measurements. Rectangular-shaped samples (width: 5.35 mm, thickness: 0.25-0.3 mm) were measured at a strain rate of 150% min^{-1}. Young's moduli were calculated from the slope in the linear region between 0 to 5 % strain.

Creep recovery. Creep recovery experiments were conducted on a TA Instruments DMA Q800 with a stress of 0.01 MPa, preload force of 0.001 MPa, and a frequency of 1 Hz, at 25 °C and 60 °C, using tensile clamps and rectangular-shaped samples (width: 5.35 mm, thickness: 0.25-0.3 mm). Tensile stress was applied for 15 min, and then the stress was removed for another 15 min.

Stress-relaxation. Stress-relaxation experiments were performed on a TA Instruments DMA Q 800 at 60 °C or 100 °C, with a constant strain rate of 1% or 60% using rectangular samples.

Optical Microscopy (OM). Images were acquired in transmittance mode on an Olympus BX41 microscope equipped with a digital camera using the Stream Basic software suite.

Synthetic Procedures and Analytical Data

Synthesis of 2-ureido-4[1H]pyrimidinone acrylate, UPyA. 2-Amino-4-hydroxy-6-methylpyrimidine (4.0 g, 0.032 mmol) was dissolved in DMSO (20 mL) in a 50 mL, one-necked round bottom flask equipped with a magnetic stirrer. The resulting mixture was heated at 170 °C under nitrogen atmosphere until complete dissolution. The oil bath was quickly removed and 2-isocyanatoethyl acrylate (4.6 mL, 0.035 mmol) was added to the flask with a syringe. The mixture was quickly cooled with an ice bath. Finally, the precipitation of **UPyA** monomer was induced by washing the solution with a large excess of acetone. The liquid suspension was filtered, and solid **UPyA** was collected and dried in vacuo at 50 °C for 24 h (7.65 g, 0.029 mmol, 91% yield). ^{1}H-NMR (400 MHz, DMSO-d$_6$): δ= 11.45 (bs, 1H), 9.77 (bs, 1H), 7.62 (bs, 1H), 6.35 (m, 1H), 6.18 (m, 1H), 5.97 (m, 1H), 5.78 (s, 1H), 4.19 (t, 2H), 3.45 (q, 2H), 3.32 (H_2O), 2.5 (DMSO), 2.1 (s, 3H); ^{13}C-NMR (100 MHz, DMSO-d$_6$): δ= 166.26, 132.73, 129.05, 63.92, 40.45.

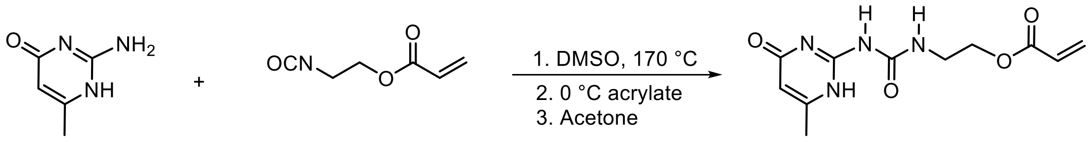

Scheme 1. Synthesis of 2-ureido-4[1H]pyrimidinone acrylate, **UPyA**.

Synthesis of PBA-UPy. UPyA (554 mg, 2.08 mmol) was added in a 20 mL one-neck Schlenk flask equipped with a magnetic stirrer and dissolved in DMSO (11.46 mL). After complete dissolution, azobisisobutyronitrile (1.81 mg, 0.011 mmol), *n*-butyl acrylate (3.0 mL, 20.83 mmol) and 2-(dodecylthiocarbonothioylthio)-2-methylpropionic acid (13.34 mg, 0.042 mmol)

were added to the mixture. An aliquot (t_0) of the solution was taken and the mixture was sparged with N_2 for 30 min. Later, the reaction was run in an inert atmosphere (Ar) at 70 °C for 2 h. The polymerization was quenched by exposure to air, by simply removing the septum. A second aliquot was taken (t_f) in order to calculate the final conversion of the monomer. The reaction mixture was precipitated by pouring it into 50 mL cold MeOH under stirring. The precipitate was redissolved in THF and dialyzed against THF for three days. The material was dried in vacuo at 50 °C for 24h and the target polymer **PBA-UPy** was obtained as yellowish solid (2.15 g, 82% yield, 74% conv., M_n = 52 kg mol^{-1}, Đ = 1.2). The reaction was repeated several times with yields of 67-82%, UPy fractions of 10 mol%, M_n = 50-54 kg mol^{-1}, and Đ = 1.2-1.3. ^{1}H-NMR (400 MHz, $CDCl_3$) of **PBA-UPy**: δ= 12.97 (bs, 1H), 11.93 (bs, 1H), 10.47 (bs, 1H), 7.29 ($CHCl_3$), 5.81 (br, 1H), 4.41-3.76 (br, 20H), 2.61-2.11 (br, 14H), 2.0-1.18 (br, 66H), 1.07-0.65 (br, 32H); ^{13}C-NMR (100 MHz, $CDCl_3$) of **PBA-UPy**: δ = 77.36, 30.97, 19.46, 14.08.

For **PBA-UPy** the final conversion was calculated by the ^{1}H-NMR spectra in $CDCl_3$, according to the following equation:

$$\text{Conv.\%} = \frac{I_0 - I_f}{I_0} \times 100$$

I_0 and I_f correspond to the normalized integrals of the vinylic proton signal in the monomers, respectively, before (t_0) and at the end (t_f) of the polymerization. For the spectra normalization, the methylene peak in alpha to the oxygen (-O**CH₂**CH₂CH₂CH₃) was used as internal standard, since its concentration remains constant during the whole polymerization, being present both in the monomer and the polymer.

The number average molecular weight (M_n) and the polydispersity Đ were obtained by SEC analysis using a calibration curve obtained by poly(methyl methacrylate) (PMMA) standards.

Preparation of PBA-UPy films. PBA-UPy (250 mg, 0.005 mmol) was suspended in $CHCl_3$ (6 mL) and stirred until dissolved. The solution was cast into a Teflon® Petri dish with a diameter of 6 cm, which was placed in a vacuum oven at 50 °C for 24 h. The samples were removed from the mold and compression-molded between Teflon® sheets in a Carver press at 60 °C and a pressure of 3 tons for 20 min to obtain 250-300 µm thick, homogeneous films.

Synthesis of MeBip-OH. Chelidamic acid (6.78 g, 37.0 mmol) and N-methyl-1,2-phenylenediamine (8.5 mL, 75.0 mmol) were added to 25 mL of phosphoric acid (85%) and stirred at 220 °C for 10 h. After removing from heat, the dark blue mixture was poured into 600 mL of water and stirred until it yielded a light blue precipitate. The precipitate that formed was filtered and then resuspended in 1 L of 10% K_2CO_3 solution. The mixture was stirred until all the solids had become pink in color, and the precipitate was once again filtered off. The solid were washed twice with water and then dissolved in a stirring flask containing 1 L of hot methanol. The dark pink methanolic solution was neutralized dropwise with 1.0 M HCl until it turned a dark blue color and then was allowed to stand and cool overnight. The product was collected as purple feathery crystals filtered from the dark blue solution, yielding 11.0 g (31.0 mmol) of 2,6-bis(10 -methyl-benzimidazolyl)-4-hydroxypyridine (MeBip-OH) (84%).^{1}H-NMR (DMSO-d_6): δ 11.36 (1H, bs), 7.79 (2H, s), 7.72 (4H, m), 7.33 (4H, m), 4.25 (6H, s); ^{13}C-NMR (DMSO-d_6): δ 165.8, 151.6, 150.5. 142.6, 137.7, 123.9, 123.1, 120.2, 113.2, 111.6 (Ar), 33.4 (CH_3).

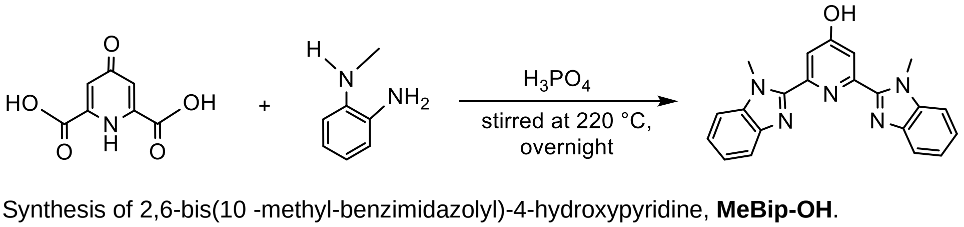

Scheme 2. Synthesis of 2,6-bis(10 -methyl-benzimidazolyl)-4-hydroxypyridine, **MeBip-OH**.

Synthesis of MeBip-C_{12}-OH. Mebip-OH (2.0 g, 5.63 mmol) was dissolved in anhydrous DMF (115 mL) in A 250 mL two-necks flask. K_2CO_3 (2.22 g, 16.05 mmol) was added, and the suspension was stirred for 20 min. The mixture was heated at 60 °C, and a solution of 12-Bromo-1-dodecanol (2.13 g, 8.03 mmol) in anhydrous DMF (10 mL) was added dropwise to the reaction mixture. The mixture was stirred for 15 h. DMF was removed under reduced pressure, and the crude redissolved in $CHCl_3$. The solution was filtered out to remove the salt, and $CHCl_3$ was evaporated under reduced pressure. The crude was re-crystallized by dissolving it in the smaller amount of CH_2Cl_2 and adding hexane dropwise to get crystals. The crystals were isolated by filtration under vacuo. The filtered solution was then allowed to stay in the freezer for 2 h to induce further crystallization and allow to isolate more crystals by filtration. This procedure was repeated twice. The product was isolated as an orange solid, 12-((2,6-Bis(1-methyl-1H-benzo[d]imidazol-2-yl)pyridin-4-yl)oxy)dodecan-1-ol (2.64 g, 4.89 mmol, 87%). ^{1}H-NMR (400 MHz, $CDCl_3$): δ = 7.86 (s, 2H), 7.80 (d, J = 6.7 Hz, 2H), 7.39 (d, J = 7.2 Hz, 2H), 7.29 (m, 4H), 4.17 (m, 8H), 3.57 (t, J = 6.6 Hz, 2H), 1.79 (q, J = 7.0 Hz, 2H), 1.59-1.18 (m, 18H). ^{13}C-NMR (101 MHz, $CDCl_3$): δ = 166.74, 150.99, 150.42, 142.39, 137.14, 123.58, 122.87, 120.12, 111.93, 109.93, 68.73, 63.02, 32.83, 32.54, 29.50, 29.42, 29.39, 29.35, 29.14, 28.88, 25.83, 25.73.

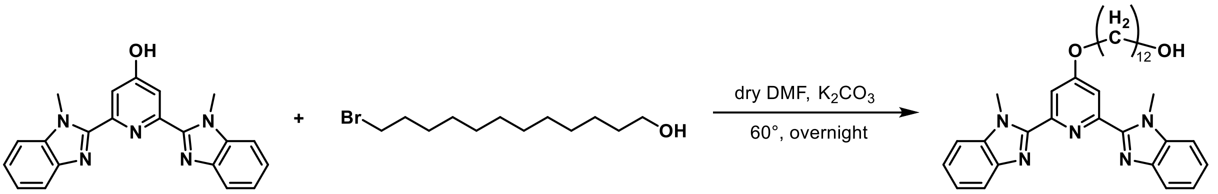

Scheme 3. Synthesis of 12-((2,6-Bis(1-methyl-1H-benzo[d]imidazol-2-yl)pyridin-4-yl)oxy)dodecan-1-ol, **MeBip-C12-OH**.

Synthesis of MeBip-A. Mebip-C_{12}-OH (2.64 g, 4.89 mmol) was dissolved in anhydrous $CHCl_3$ (135 mL) and the solution was cooled in an ice bath. Triethylamine (2.8 mL, 20 mmol) was added dropwise and the solution was stirred for 30 min. Acryloyl chloride (485 mL, 5.96 mmol) was added dropwise at 0°C, and the reaction mixture was allowed to warm to room temperature and stirred for 17 h. $CHCl_3$ was removed under reduced pressure. The crude was suspended in THF, and the solution was filtered off. The solvent was removed and the crude was redissolved in CH_2Cl_2. The solution was washed two times with $NaHCO_3$, and once with brine. The organic phase was dried over Na_2SO_4, and the solvent was removed in vacuo. Column chromatography (silica gel, DCM/MeOH 98:2–97:3 v/v) yielded the target compound as a light yellow solid (2.0 g, 3.4 mmol, 70 %). ^{1}H-NMR (400 MHz, $CDCl_3$): δ = 7.96 (s, 2H), 7.84 (d, J = 7.03 Hz, 2H), 7.41 (d, J = 7.2 Hz, 2H), 7.32 (m, 4H), 6.31 (dd, J = 17.3 Hz, J = 1.5 Hz, 1H), 6.04 (dd, J = 17.3 Hz, J = 10.4 Hz, 1H), 5.73 (dd, J = 10.4 Hz, J = 10.5 Hz, J = 1.5 Hz, 1H), 4.19 (m, 8H), 4.07 (t, J = 6.8 Hz, 2H), 1.79 (q, J = 7.0 Hz, 2H), 1.58 (q, J = 7.0 Hz, 2H), 1.41 (q, J = 7.3 Hz, 2H), 1.34-1.15 (m, 14H). ^{13}C-NMR (101 MHz, $CDCl_3$): δ = 166.91, 166.49, 150.48, 137.23, 130.51, 128.83, 123.79, 123.09, 120.23, 112.18, 110.10, 77.36, 68.94, 64.89, 32.70, 29.69, 29.65, 29.41, 29.05, 28.78, 26.09, 26.03.

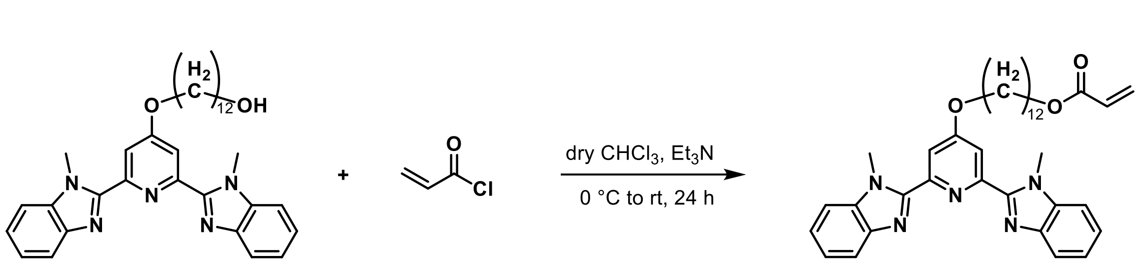

Scheme 4. Synthesis of **MeBipA**.

Synthesis of PBA-MeBip. MeBipA (232 mg, 0.39 mmol) was added in a 5 mL one-neck Schlenk flask equipped with a magnetic stirrer and dissolved in DMF (1.5 mL). After complete dissolution, azobisisobutyronitrile (2.15 mg, 0.013 mmol), *n*-butyl acrylate (1.12 mL, 7.8 mmol) and 2-(dodecylthiocarbonothioylthio)-2-methylpropionic acid (7.9 mg, 0.025 mmol) were added to the mixture. An aliquot (t_0) of the solution was taken, and the mixture was sparged with N_2 for 30 min. The reaction was run in an inert atmosphere (Ar) at 70 °C for 2 h. The polymerization was quenched by exposure to air, by simply removing the septum. A second aliquot was taken (t_f) in order to calculate the final conversion of the monomer. The reaction mixture was precipitated by pouring it into 10 mL cold MeOH under stirring. After removing the solvent by decantation, the precipitate was redissolved in THF and dialyzed against THF for three days. The material was dried in vacuo at 100 °C for 24h and the target polymer **PBA-MeBip** was obtained as yellowish solid (500 mg, 45% yield, 63% conv., M_n = 30 kg mol^{-1}, Đ = 1.2). The reaction was repeated several times with yields of 40-61%, MeBip fractions of 5 mol%, M_n = 30-34 kg mol^{-1}, and Đ = 1.2-1.3. ^{1}H-NMR (400 MHz, $CDCl_3$) of **PBA-MeBip**: δ = 7.86 (br, 2H), 7.79 (br, 2H), 7.39 (br, 2H), 7.29 (br, 4H), 4.17 (br, 8H), 4.07-3.83 (br, 40), 2.35-2.10 (br, 20H), 1.92-1.14 (br, 170H), 0.93-0.75 (br, 60H). ^{13}C-NMR (100 MHz, $CDCl_3$) of **PBA-MeBip**: δ = 174.55, 151.51, 135.78, 128.25, 125.51, 119.88, 110.05, 67.97, 64.41, 41.50, 34.23, 32.62, 30.63, 30.60, 30.33, 29.64, 25.93, 25.62, 19.10, 13.73.

For **PBA-MeBip** the final conversion was calculated by the ^{1}H-NMR spectra in $CDCl_3$, according to the following equation:

$$\text{Conv.\%}= \frac{I_0 - I_f}{I_0} \times 100$$

I_0 and I_f correspond to the normalized integrals of the vinylic proton signal in the monomers, respectively, before (t_0) and at the end (t_f) of the polymerization. For the spectra normalization, the methylene peak in alpha to the oxygen (-O**CH_2**$CH_2CH_2CH_3$) was used as internal standard, since its concentration remains constant during the whole polymerization, being present both in the monomer and the polymer.

The number average molecular weight (M_n) and the polydispersity Đ were obtained by SEC analysis using a calibration curve obtained by poly(methyl methacrylate) (PMMA) standards.

Preparation of PBA-MeBip·Zn^{2+} films. PBA-MeBip (250 mg, 0.008 mmol) was suspended in $CHCl_3$ (6 mL) and stirred until dissolved. Then, a solution of $Zn(OTf_2)_2$ (50.0 mg, 0.14 mmol) in anhydrous CH_3CN/MeOH (9:1) (4 mL) was added, and an increase of the solutions viscosity was observed. The solution mixture was stirred for 30 min and then cast into a Teflon® Petri dish with a diameter of 6 cm, which was placed in a vacuum oven at 100 °C for 24 h. The samples were removed from the mold and compression-molded between Teflon® sheets in a Carver press at 120 °C and a pressure of 3 tons for 20 min to obtain 250-300 µm thick, homogeneous films.

Synthesis of DN. The double network **DN** was prepared by mixing the polymer solution of **PBA-UPy** (250 mg, 0.005 mmol) in 6 mL of $CHCl_3$ and **PBA-MeBip** (250 mg, 0.008 mmol) in 6 mL of $CHCl_3$, to obtain a homogeneous solution. A solution of $Zn(OTf_2)_2$ (50.0 mg, 0.14 mmol) in anhydrous CH_3CN (4 mL) was added under stirring, and an increase of the solutions viscosity was observed. The solution mixture was stirred for 30 min and then cast into a Teflon® Petri dish with a diameter of 6 cm, which was placed in a vacuum oven at 100 °C for 24 h. The samples were removed from the mold and compression-molded between Teflon® sheets in a Carver press at 120 °C and a pressure of 3 tons for 20 min to obtain 250-300 µm thick, homogeneous films.

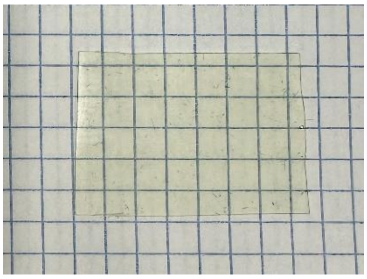

Figure S1. Picture of a film of **DN**.

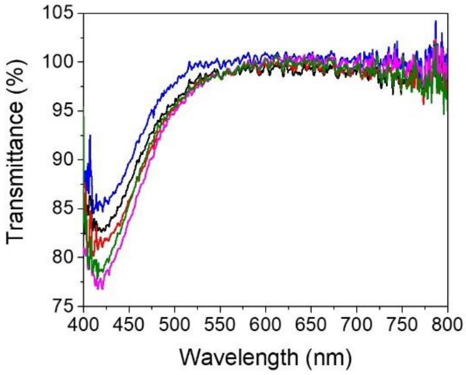

Figure S2. Transmission measurements of **DN**. The different traces represent five individual measurements of diverse areas of **DN** surface (sample thickness= 250 μm). Measurements were calibrated using a glass slide as the light reference, while the dark reference was recorded with the light source covered with a black sheet.

TGA Data

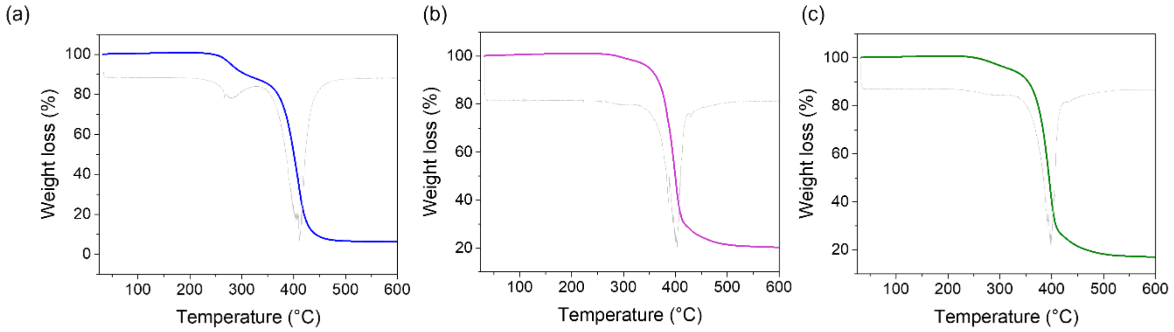

Figure S3. Thermal gravimetric analysis (TGA) traces and derivatives, measured in the range 25 - 600 °C with a heating rate of 10 °C min^{-1} of **PBA-UPy** (blue), **PBA-MeBip·Zn^{2+}** (purple), **DN** (green).

DSC Data

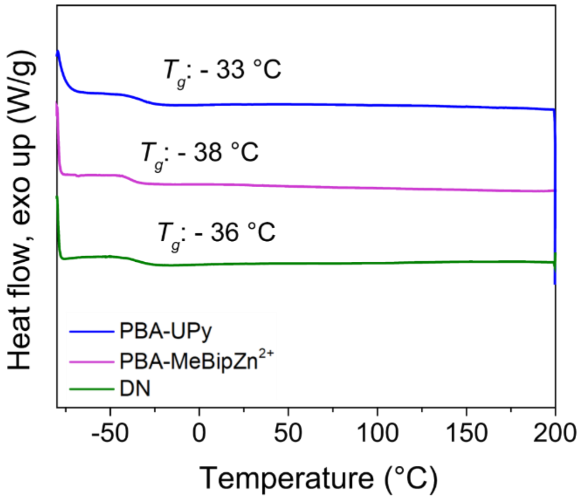

Figure S4. Differential scanning calorimetry (DSC) traces, measured in the range -80 – 200 °C with a heating rate of 10 °C min^{-1}, 2nd heating, of **PBA-UPy** (blue), **PBA-MeBip·Zn^{2+}** (purple), **DN** (green).

X-Ray Scattering of DN

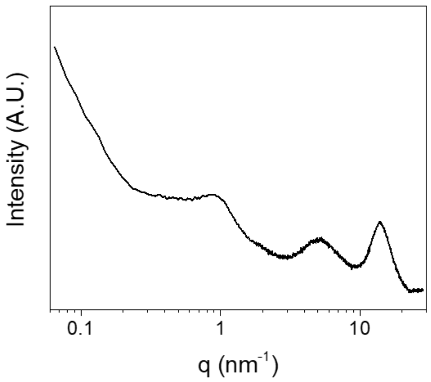

Figure S5. X-Ray scattering (SAXS-WAXS analysis) of **DN**.

Creep recovery experiment

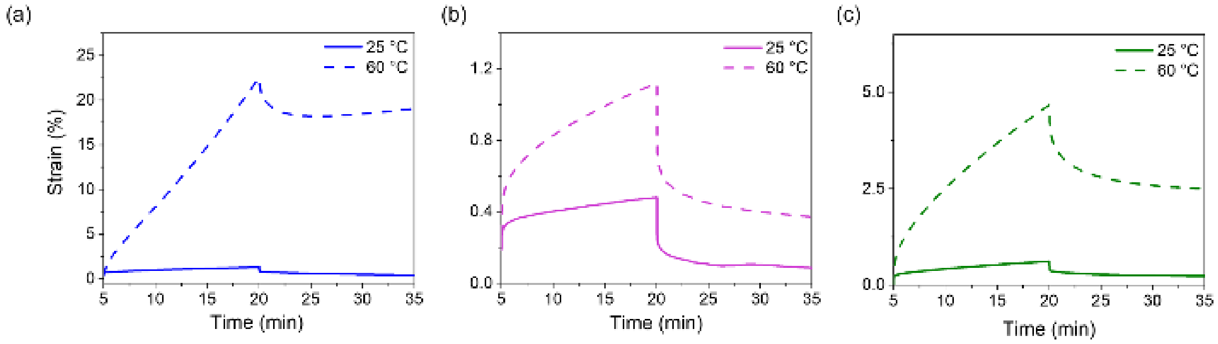

Figure S6. Creep recovery experiments of **PBA-UPy** (a, blue), **PBA-MeBip·Zn^{2+}** (b, purple) and **DN** (c, green) at 25 °C (solid lines) and 60 °C (dashed lines), performed with a constant load of 0.01 MPa for 15 min. After 15 min, the stress was released.

Stress-relaxation experiments

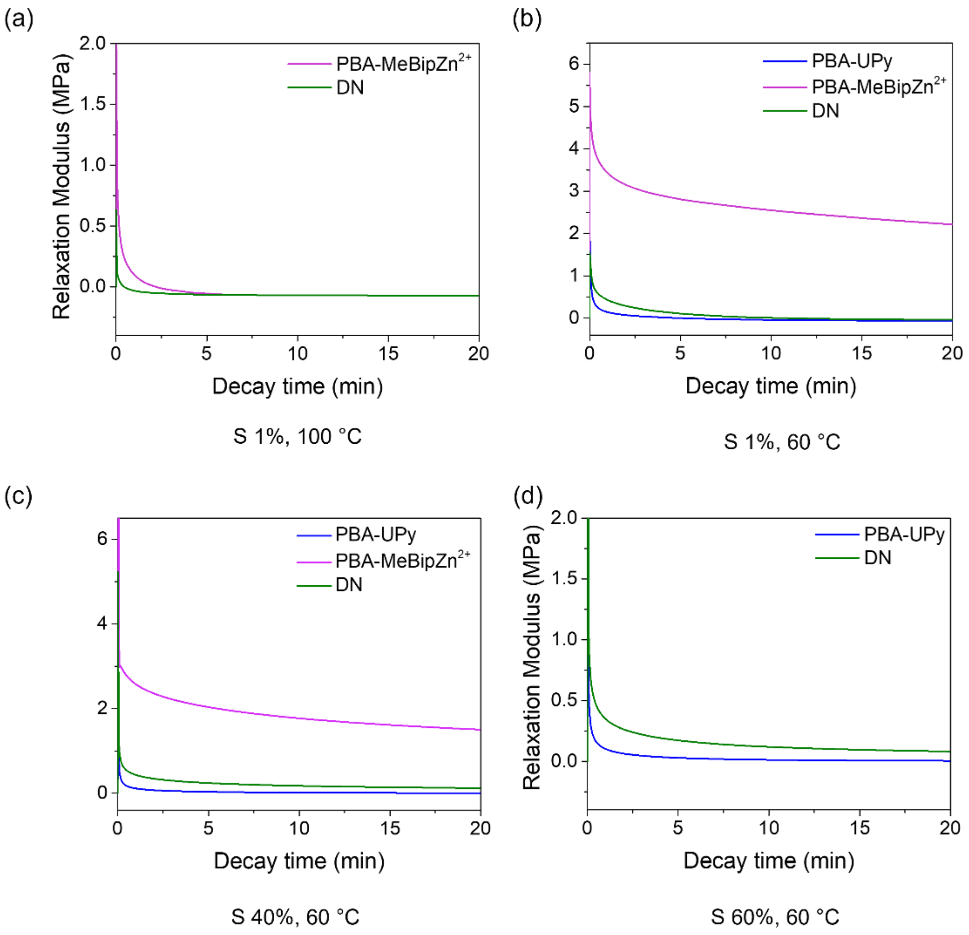

Figure S7. Stress-relaxation experiments of **PBA-UPy** (blue), **PBA-MeBip·Zn^{2+}** (purple), **DN** (green). The analyses were conducted at 60 °C or 100 °C, with a constant strain of 1%, 40% or 60%.

Thermo-chemical healing experiments

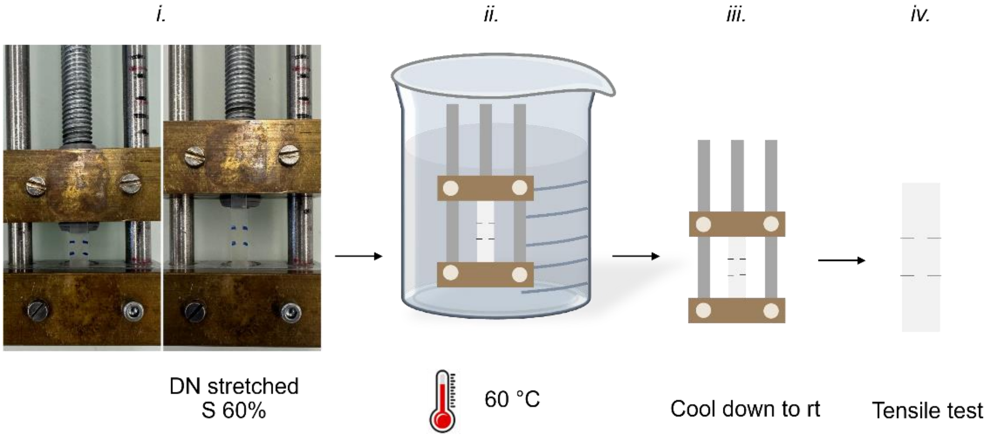

Figure S8. Pre-stretching of the **DN**. (*i*) First, a strain of 60% was applied to a strip of the **DN** under ambient conditions. This was done in a mechanical stretching device as shown. (*ii*) The sample (still mounted in the stretcher) was then placed into a water bath that was heated to 60 °C for 10 minutes to pre-stretch the **PBA-MeBip·Zn^{2+}** network. (*iii*) The sample was removed from the water bath and allowed to cool to room temperature (still mounted in the stretcher). (*iv*) Finally, the sample was removed from the stretcher, and uniaxial tensile tests were carried out.

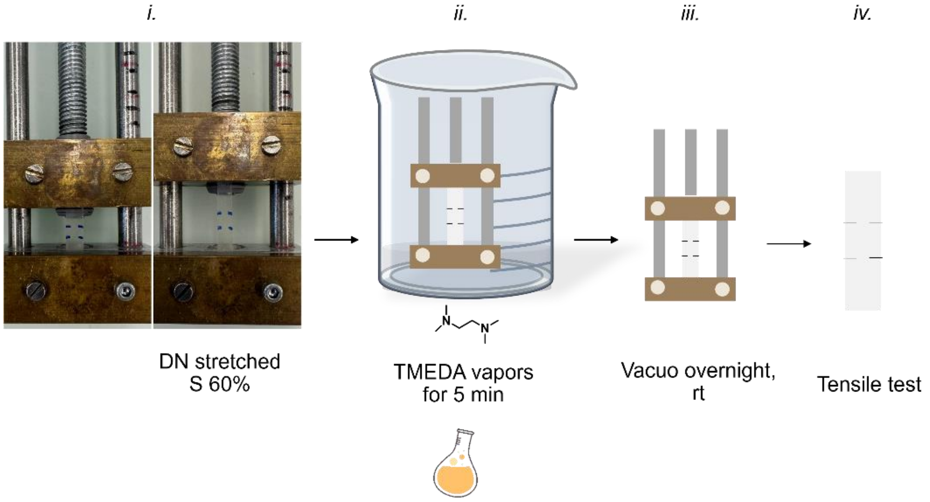

Figure S9. (*i*) Pre-stretching of the **DN**. (*i*) First, a strain of 60% was applied to a strip of the **DN** under ambient conditions. This was done in a mechanical stretching device as shown. (ii) The sample (still mounted in the stretcher) was exposed to TMEDA vapors for 5 minutes to pre-stretch the **PBA-UPy** network. (*iii*) The sample was placed in vacuo at room temperature to remove TMEDA traces (still mounted in the stretcher). (*iv*) Finally, the sample was removed from the stretcher, and uniaxial tensile tests were carried out.

Thermo-chemical healing experiments

Films were damaged by cutting the samples **PBA-UPy**, **PBA-MeBip·Zn^{2+}** and **DN** to a depth of ~50 % of their thickness with a razor blade attached to a caliper, which allowed for specific depth control. The samples were then exposed to heat (90 °C or 120 °C for different exposure time) or to TMEDA vapors. The healing efficiency (%) was calculated according to the equation:

$$\%\,Healing\ efficiency = \frac{Toughness\ healed}{Toughness\ as\ prepared} \cdot 100$$

Table S1. Mechanical properties (established by tensile tests) of supramolecular polymer network **PBA-UPy**, **PBA-MeBip·Zn^{2+}** and supramolecular double network **DN.**[a]

	Young's Modulus[b] E_t (MPa)	Stress at break[b] σ_γ (MPa)	Elongation at break[b] e_β (%)	Toughness[c] (KJm^{-3})	Healing efficiency[d] (%)
**PBA-UPy as prepared**	2.9 ± 0.2	2.3 ± 0.2	109 ± 10.5	1230 ± 252	-
**PBA-UPy scratched**	3.2 ± 0.1	1.4 ± 0.2	62 ± 2.0	660 ± 70	-
**PBA-UPy healed 90 °C, 40 min**	2.9 ± 0.3	2.0 ± 0.3	98 ± 14	1001 ± 234	81 ± 11
**PBA-MeBip·Zn^{2+} as prepared**	9.0 ± 0.3	2.9 ± 0.3	91 ± 12	1646 ± 470	-
**PBA-MeBip·Zn^{2+} scratched**	6.0 ± 0.3	1.1 ± 0.1	35 ± 6.3	225 ± 63	79 ± 13
**PBA-MeBip·Zn^{2+} TMEDA, 5 min**	9.3 ± 0.4	2.6 ± 0.1	76 ± 8.0	1299 ± 230	-
**DN as prepared**	4.9 ± 0.2	2.3 ± 0.2	180 ± 20	2814 ± 930	-
**DN scratched**	4.3 ± 0.1	1.2 ± 0.1	61 ± 8.1	448 ± 110	-
**DN 120 °C, 90 min**	4.8 ± 0.2	2.7 ± 0.2	128 ± 10	1892 ± 247	67 ± 19
**DN TMEDA, 5 min**	4.8 ± 1.0	2.2 ± 0.3	117 ± 12	1615 ± 236	57 ± 15
**DN 120 °C, 90 min & TMEDA, 5 min**	4.8 ± 0.3	2.3 ± 0.2	170 ± 20	2648 ± 504	94 ± 23

[a]Data represent averages of n = 3−8 individual measurements ± standard deviation. [b]Measured by stress−strain experiments at 25 °C with a strain rate of 150%/min. The Young's modulus was calculated from the slope in the linear region between 0 to 5 % strain. [c]The toughness was calculated from the area under the stress-strain curve. [d]Calculated by the ratio between the toughness of healed and as prepared material.

Tensile data of PBA-UPy, PBA-MeBip·Zn^{2+} and DN

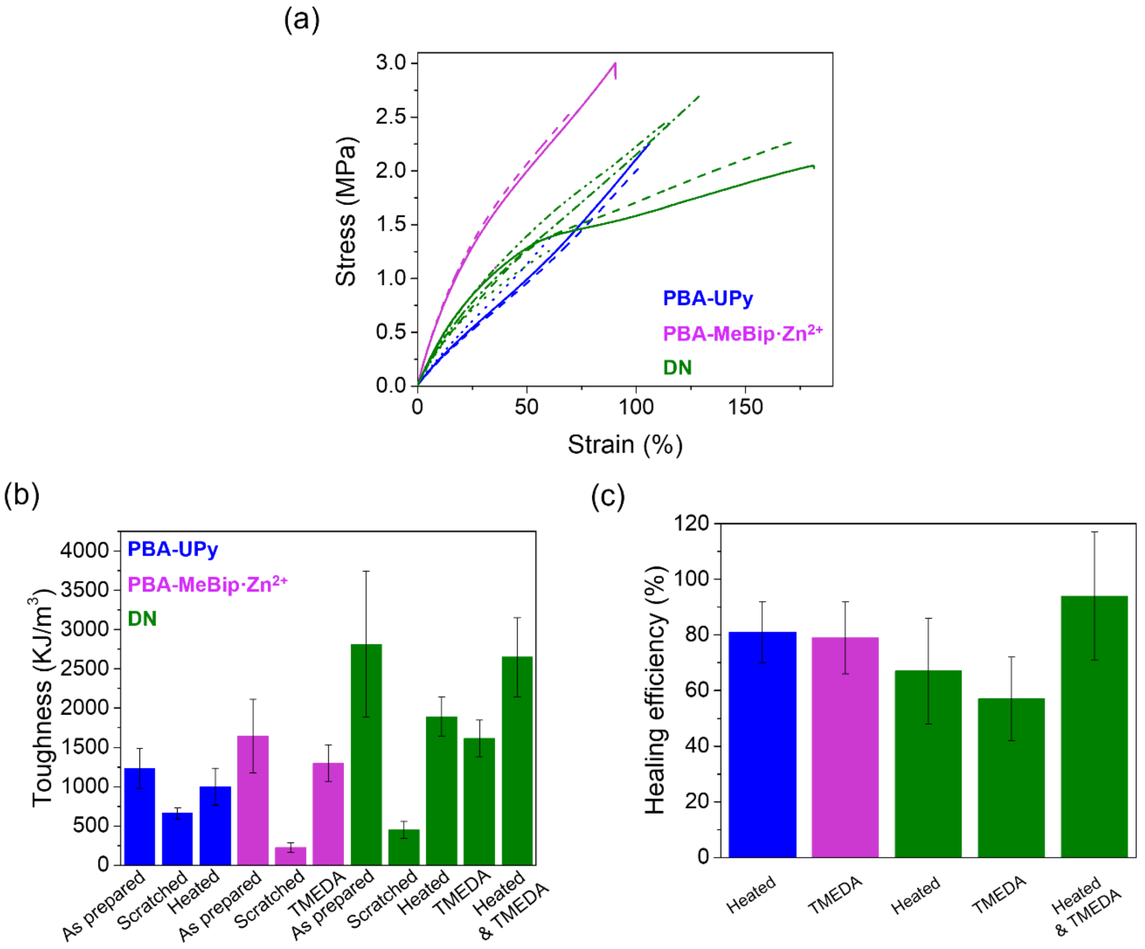

Figure S10. (a) Stress-strain curves of as prepared (solid lines), scratched (dotted lines) and healed (dashed lines and dashed-dotted lines) films of **PBA-UPy**, **PBA-MeBip·Zn^{2+}** and **DN.** All films were scratched to a depth of 50% and healed at either 90 °C for 40 minutes (**PBA-UPy**), at 120 °C for 90 min (**DN**), or exposed to TMEDA vapors for 5 minutes (**PBA-MeBip·Zn^{2+}**) .(b) Toughness of the samples shown in (a) expressed by the area under the stress-strain curves shown in (a). (c) Healing efficiency achieved for the different materials and protocols. For the data shown in a-c, the films had been cut to a depth of 50%. All the error bars represent the standard deviation.

UV-Vis spectra

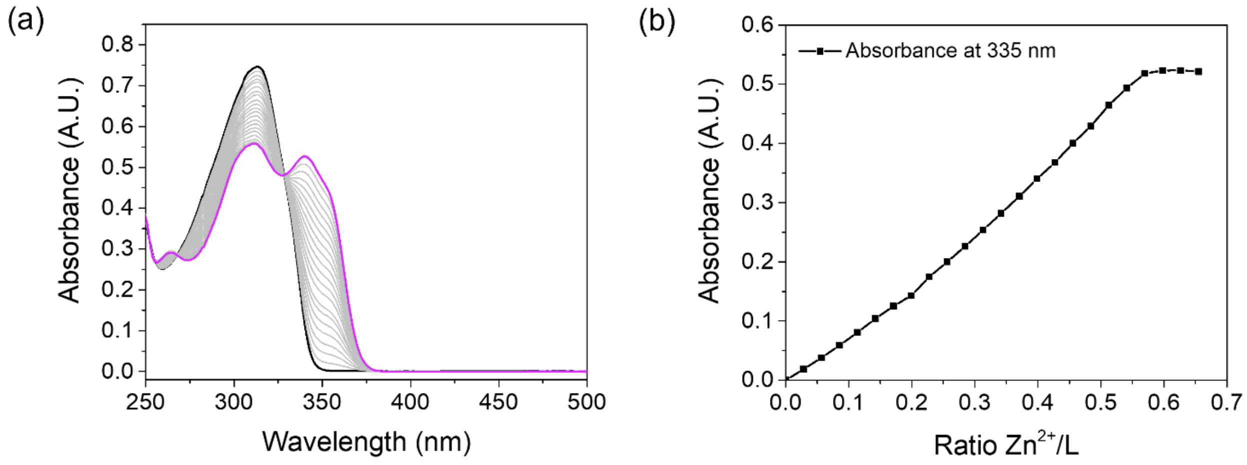

Figure S11. a) UV-Vis absorption spectra recorded for the titration of a solution of **PBA-MeBip** with $Zn(NTf_2)_2$. Data were acquired upon adding 25 µL aliquots of a $Zn(NTf_2)_2$ solution in a 9:1 v/v $CHCl_3/CH_3CN$ mixture to a solution of **PBA-MeBip** in the same solvent mixture. The traces show the UV-Vis spectra of **PBA-MeBip** (black, neat polymer) and **PBA-MeBip·Zn^{2+}** (purple, fully complexed). b) Absorbance of **PBA-MeBip** in a 9:1 v/v $CHCl_3/CH_3CN$ mixture at 335 nm at various concentrations of Zn^{2+}.

NMR Spectra

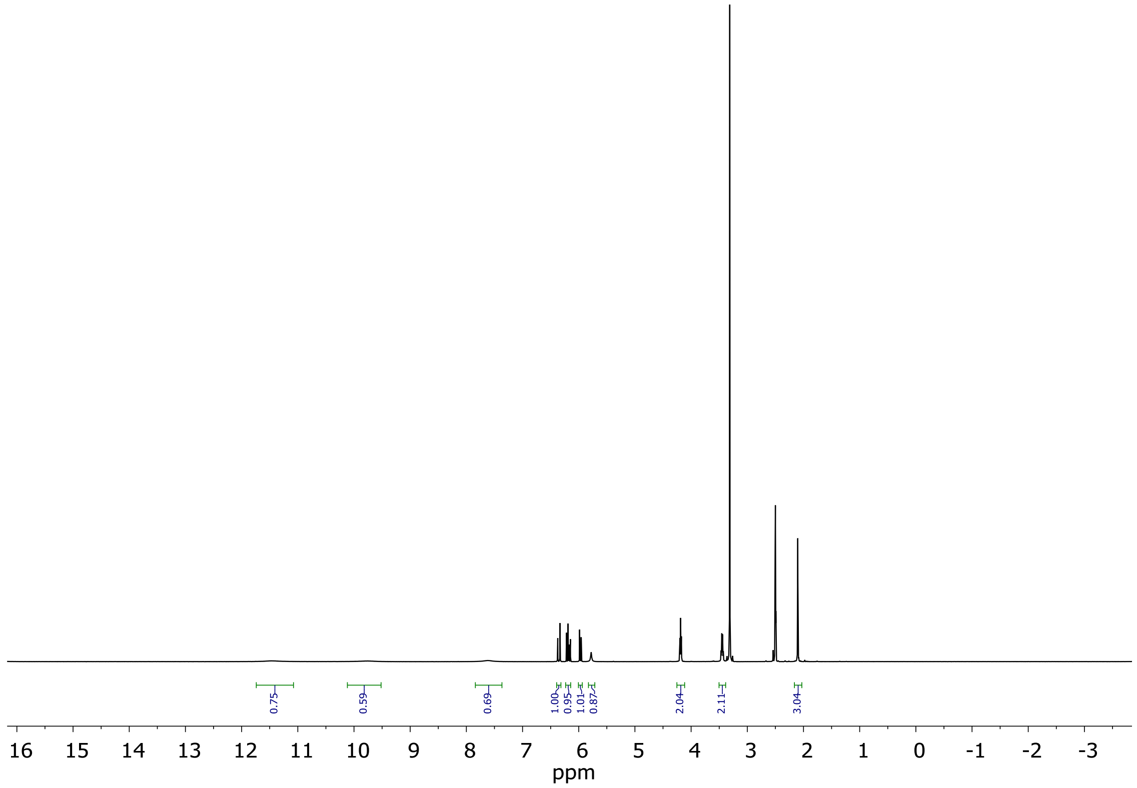

Figure S12. ^{1}H-NMR (DMSO-d_6, 400 MHz) spectrum of 2-ureido-4[1H]pyrimidinone acrylate **UPyA**.

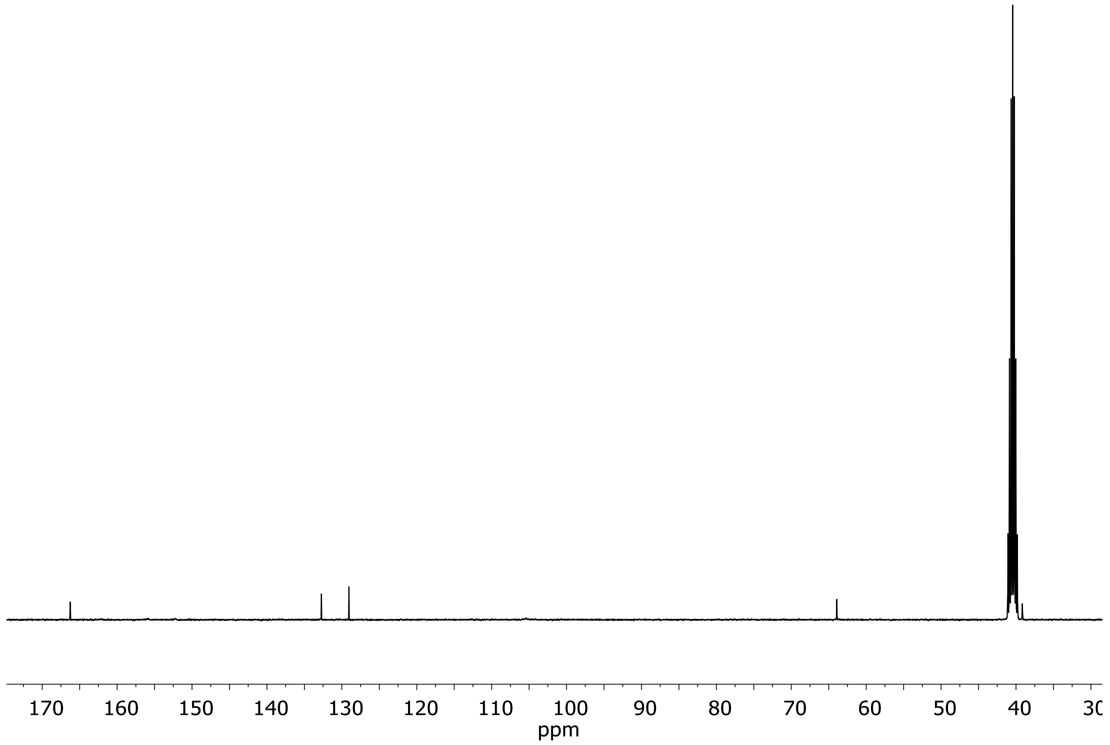

Figure S13. ^{13}C-NMR (DMSO-d_6, 100 MHz) spectrum of 2-ureido-4[1H]pyrimidinone acrylate **UPyA**.

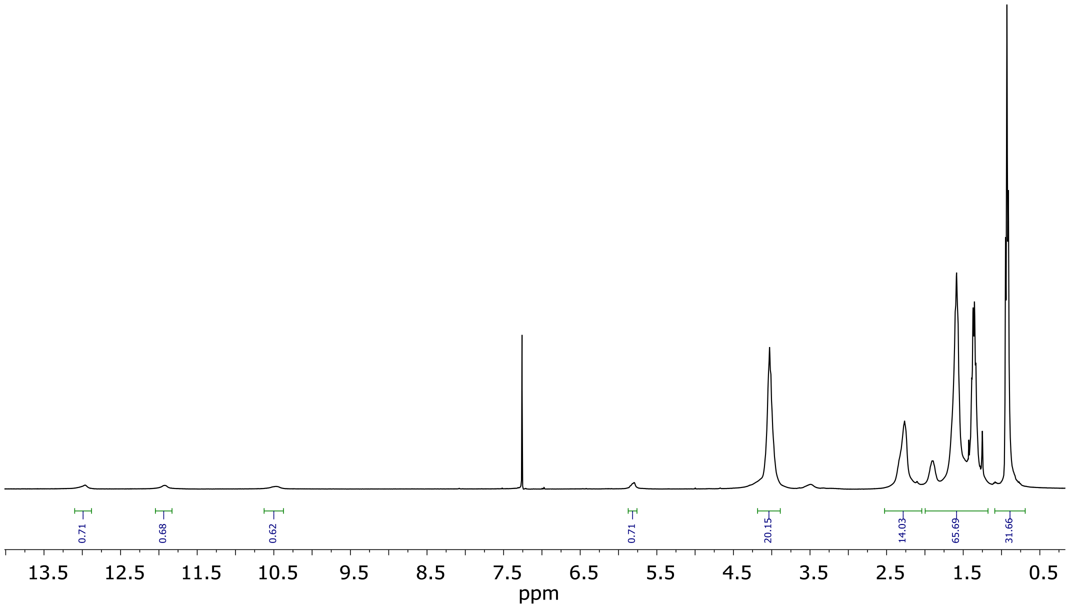

Figure S14. ^{1}H-NMR ($CDCl_3$, 400 MHz) spectrum of **PBA-UPy**.

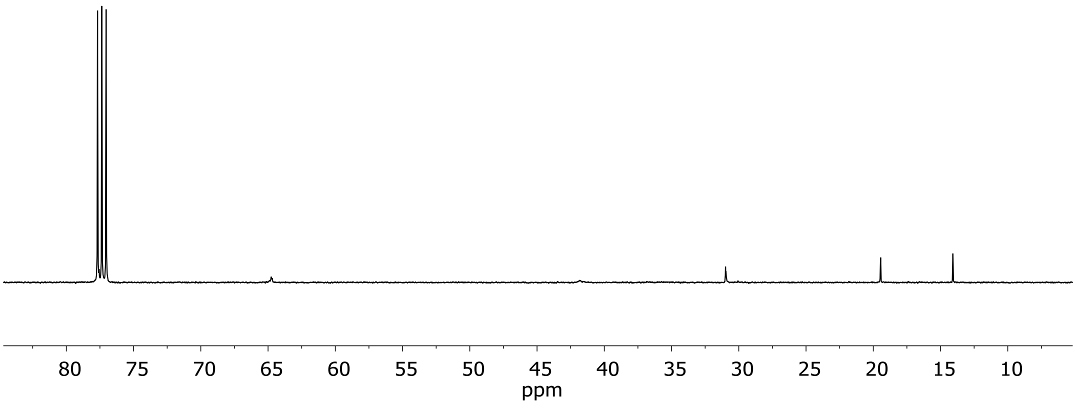

Figure S15. ^{13}C-NMR ($CDCl_3$, 100 MHz) spectrum of **PBA-UPy**.

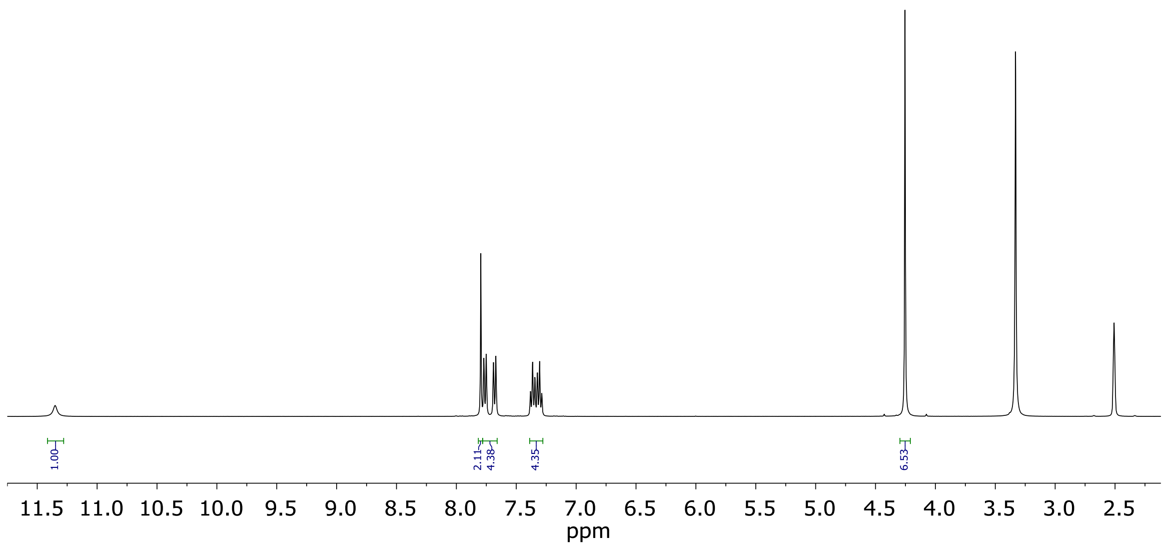

Figure S16. ^{1}H-NMR (DMSO-d_6, 400 MHz) spectrum of 2,6-bis(10-methyl-benzimidazolyl)-4-hydroxypyridine **MeBip-OH**.

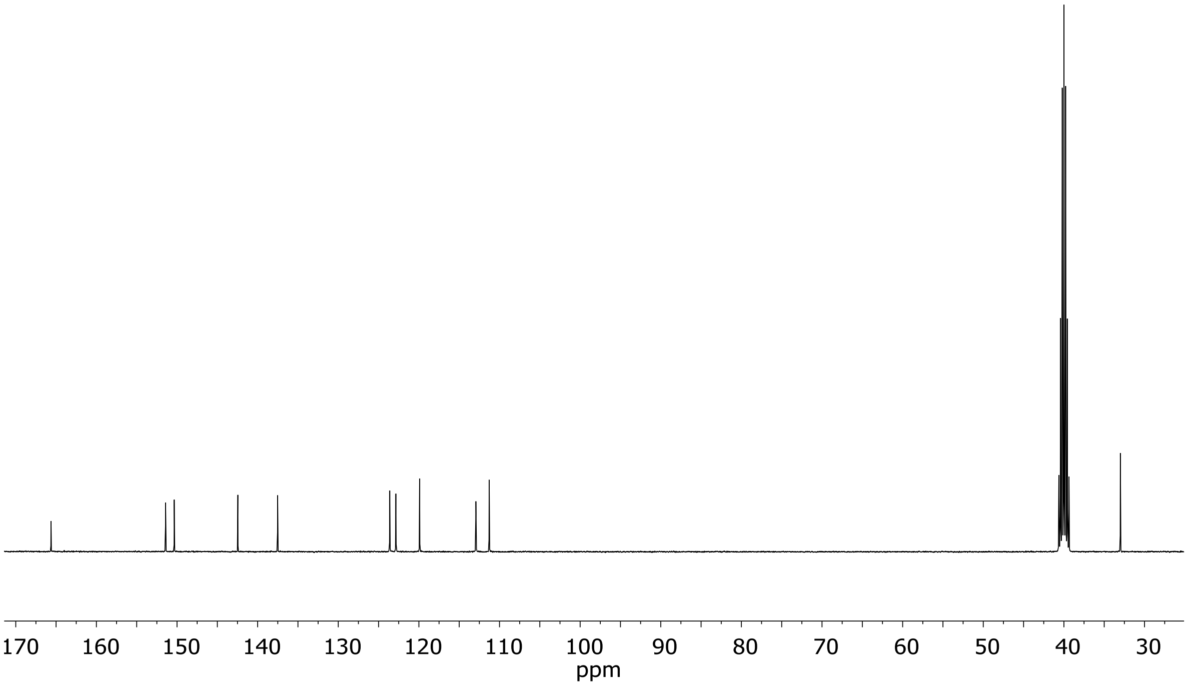

Figure S17. ^{13}C-NMR (DMSO-d_6, 100 MHz) spectrum of 2,6-bis(10-methyl-benzimidazolyl)-4-hydroxypyridine **MeBip-OH**.

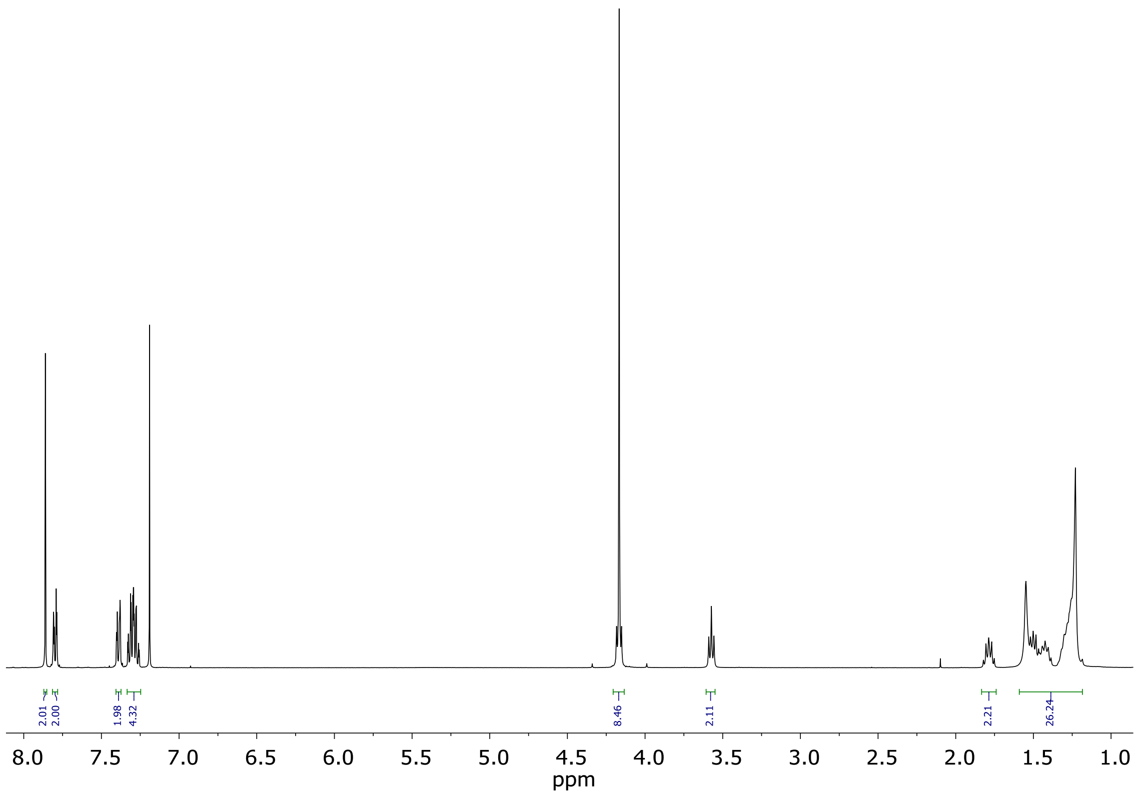

Figure S18. 1^{1}H-NMR ($CDCl_3$, 400 MHz) spectrum of 12-((2,6-bs(1-methyl-1H-benzo[d]imidazol-2-yl)pyridin-4-yl)oxy)dodecan-1-ol **MeBip-C$_{12}$-OH**.

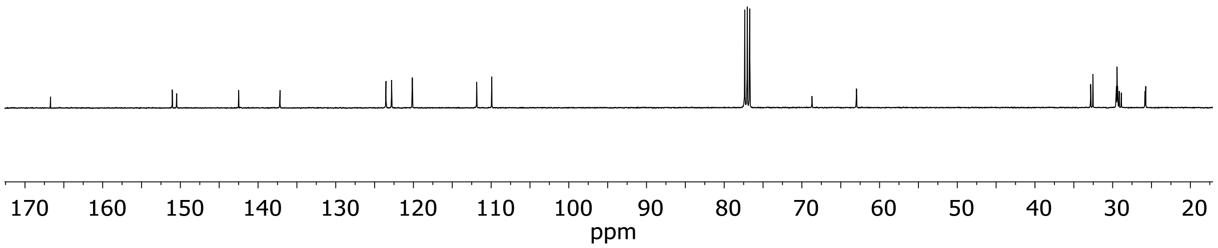

Figure S19. ^{13}C-NMR ($CDCl_3$, 100 MHz) spectrum of 12-((2,6-bis(1-methyl-1H-benzo[d]imidazol-2-yl)pyridin-4-yl)oxy)dodecan-1-ol **MeBip-C$_{12}$-OH**.

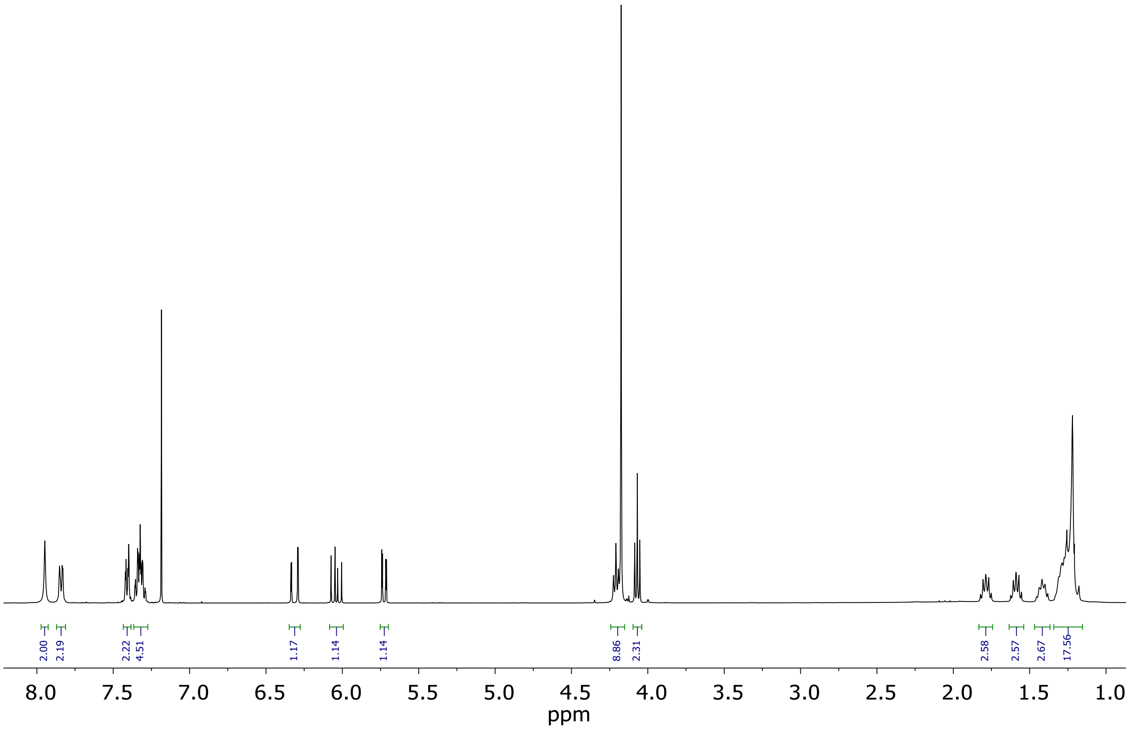

Figure S20. ^{1}H-NMR ($CDCl_3$, 400 MHz) spectrum of **MeBipA**.

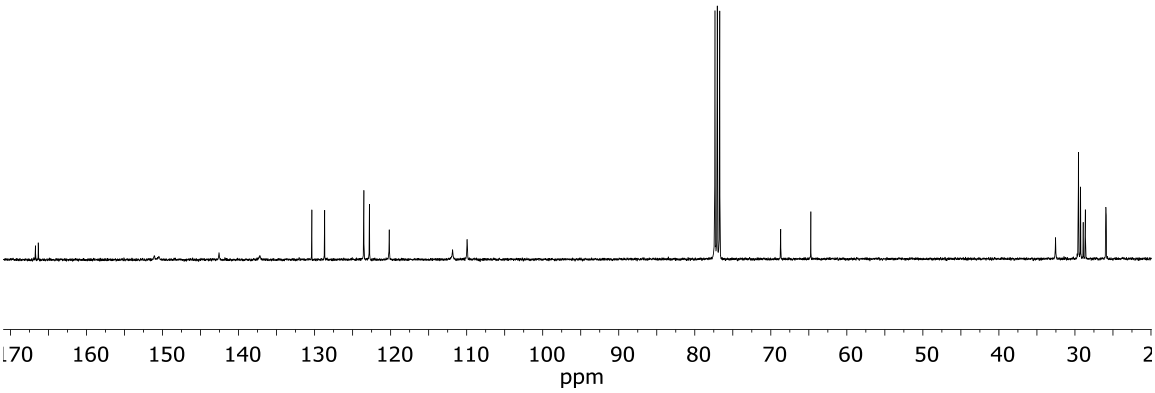

Figure S21. ^{13}C-NMR ($CDCl_3$, 100 MHz) spectrum of **MeBipA**.

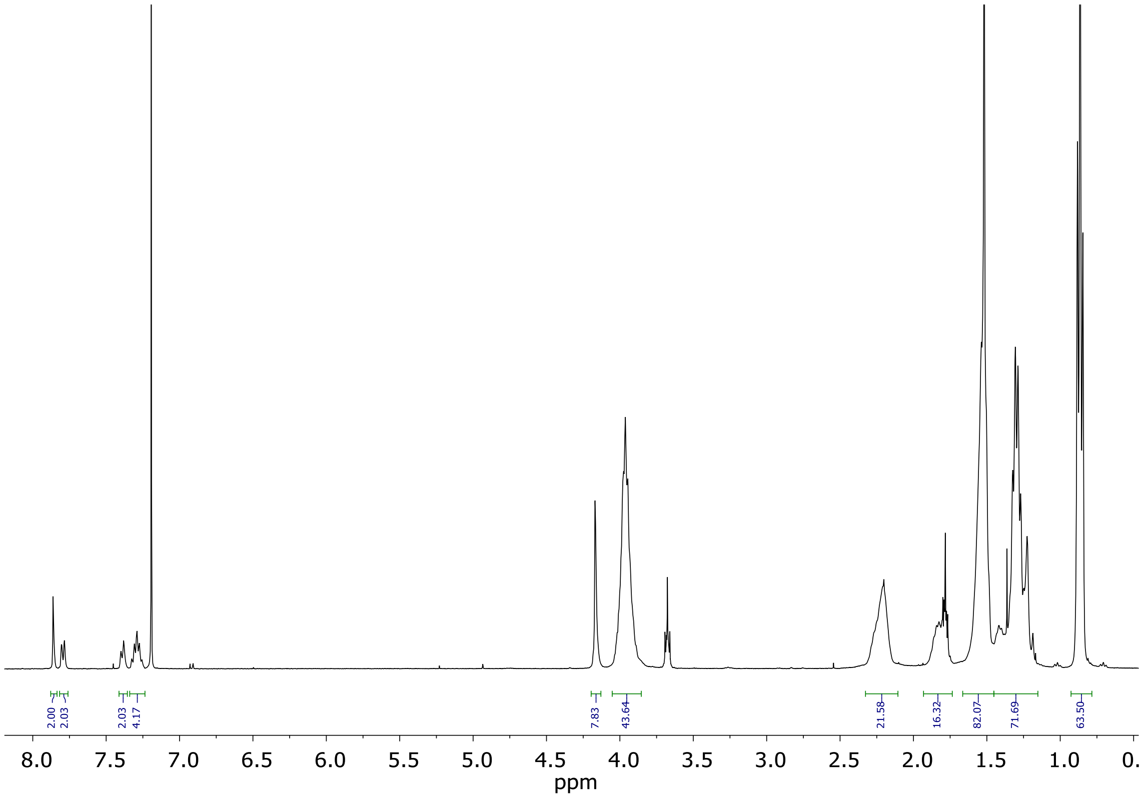

Figure S22. ^{1}H-NMR ($CDCl_3$, 400 MHz) spectrum of **PBA-MeBip.**

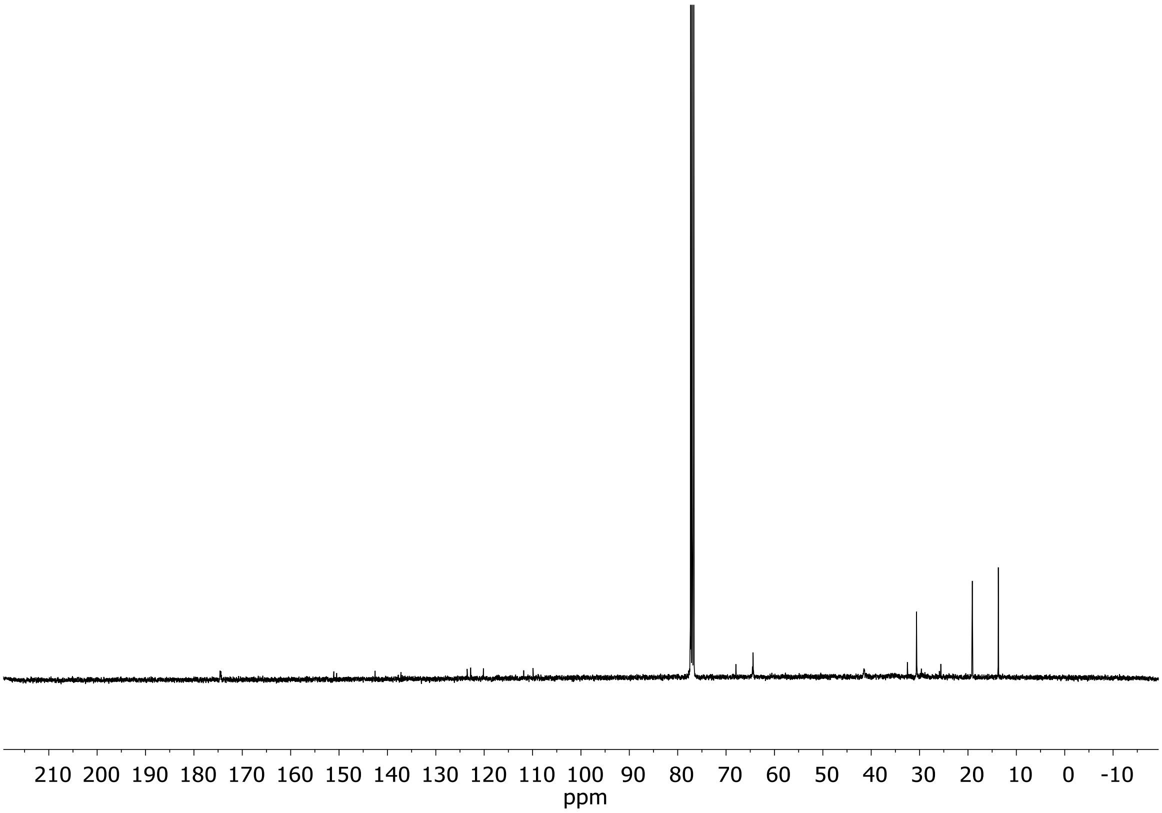

Figure S23. ^{13}C-NMR ($CDCl_3$, 100 MHz) spectrum of **PBA-MeBip.**
